# Identifying squalene epoxidase as a metabolic vulnerability in high‐risk osteosarcoma using an artificial intelligence‐derived prognostic index

**DOI:** 10.1002/ctm2.1586

**Published:** 2024-02-19

**Authors:** Yongjie Wang, Xiaolong Ma, Enjie Xu, Zhen Huang, Chen Yang, Kunpeng Zhu, Yang Dong, Chunlin Zhang

**Affiliations:** ^1^ Department of Orthopaedic Surgery Shanghai Tenth People's Hospital, School of Medicine, Tongji University Shanghai P. R. China; ^2^ Institute of Bone Tumor Affiliated to Tongji University School of Medicine Shanghai P. R. China; ^3^ Proteomics and Cancer Cell Signaling Group, German Cancer Research Center (DKFZ) Heidelberg Germany; ^4^ State Key Laboratory of Oncogenes and Related Genes Shanghai Cancer Institute, Renji Hospital, Shanghai Jiao Tong University School of Medicine Shanghai P. R. China; ^5^ Department of Orthopaedics Shanghai Jiao Tong University Affiliated Sixth People's Hospital Shanghai Jiao Tong University Shanghai P. R. China

**Keywords:** machine learning, osteosarcoma, prognostic model, squalene epoxidase

## Abstract

**Background:**

Osteosarcoma (OSA) presents a clinical challenge and has a low 5‐year survival rate. Currently, the lack of advanced stratification models makes personalized therapy difficult. This study aims to identify novel biomarkers to stratify high‐risk OSA patients and guide treatment.

**Methods:**

We combined 10 machine‐learning algorithms into 101 combinations, from which the optimal model was established for predicting overall survival based on transcriptomic profiles for 254 samples. Alterations in transcriptomic, genomic and epigenomic landscapes were assessed to elucidate mechanisms driving poor prognosis. Single‐cell RNA sequencing (scRNA‐seq) unveiled genes overexpressed in OSA cells as potential therapeutic targets, one of which was validated via tissue staining, knockdown and pharmacological inhibition. We characterized changes in multiple phenotypes, including proliferation, colony formation, migration, invasion, apoptosis, chemosensitivity and in vivo tumourigenicity. RNA‐seq and Western blotting elucidated the impact of squalene epoxidase (*SQLE*) suppression on signalling pathways.

**Results:**

The artificial intelligence‐derived prognostic index (AIDPI), generated by our model, was an independent prognostic biomarker, outperforming clinicopathological factors and previously published signatures. Incorporating the AIDPI with clinical factors into a nomogram improved predictive accuracy. For user convenience, both the model and nomogram are accessible online. Patients in the high‐AIDPI group exhibited chemoresistance, coupled with overexpression of *MYC* and *SQLE*, increased mTORC1 signalling, disrupted PI3K–Akt signalling, and diminished immune infiltration. ScRNA‐seq revealed high expression of *MYC* and *SQLE* in OSA cells. Elevated *SQLE* expression correlated with chemoresistance and worse outcomes in OSA patients. Therapeutically, silencing *SQLE* suppressed OSA malignancy and enhanced chemosensitivity, mediated by cholesterol depletion and suppression of the FAK/PI3K/Akt/mTOR pathway. Furthermore, the SQLE‐specific inhibitor FR194738 demonstrated anti‐OSA effects in vivo and exhibited synergistic effects with chemotherapeutic agents.

**Conclusions:**

AIDPI is a robust biomarker for identifying the high‐risk subset of OSA patients. The SQLE protein emerges as a metabolic vulnerability in these patients, providing a target with translational potential.

## Graphical abstract

1. AIDPI is a robust biomarker for stratifying high‐risk OSA patients characterized by dysregulated cholesterol homeostasis, and SQLE is a metabolic vulnerability of these patients.
2. Silencing *SQLE* impedes OSA by reducing cholesterol and inhibiting the FAK/PI3K/Akt/mTOR pathway.
3. SQLE inhibitor suppresses OSA progression in vivo and enhances chemotherapy efficacy.

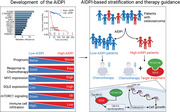

## 1 INTRODUCTION

Osteosarcoma (OSA), the most prevalent primary malignant bone tumour, is notorious for its aggressive nature, and it predominantly affects adolescents and individuals over 60 years of age.^1^ Despite advancements in surgical techniques and the adoption of the MAP regimen, consisting of high‐dose methotrexate (MTX), adriamycin (ADM), and cisplatin (DDP), high‐grade OSA has shown persistent chemoresistance, so outcomes have not markedly improved since the 1980s.^2^ Although several chemoresistant cell models have been established in vitro and various differentially expressed messenger RNAs (mRNAs) and noncoding RNAs have been identified,^3^, ^4^ new drugs with translational potential remain limited. These challenges drove us to investigate the real‐world variations among OSA patients with differing outcomes and the intrinsic mechanisms to identify therapeutic targets for those with a dismal prognosis. This initiative underscores the critical need for advanced biomarkers to identify high‐risk patients potentially resistant to standard treatments but who might benefit from additional therapies, thereby paving the way for more personalized treatment.

In clinical practice, factors such as Musculoskeletal Tumor Society (MSTS) stage III and an axial skeletal location for the primary tumour are prominent pretreatment indicators associated with unfavourable outcomes.^5^ Nevertheless, the standard chemotherapy strategy remains unchanged, irrespective of the MSTS stage or primary tumour location.^1^ Unfavourable outcomes also stem from incomplete surgical resection and a poor response to chemotherapy.^6^ The Huvos grading system quantifies the response to neoadjuvant chemotherapy by assessing the tumour necrosis rate in surgically resected samples.^7^ In this system, grade I denotes 0%–49% necrotic cells, grade II denotes 50%–89% necrotic cells, grade III denotes 90%–99% necrotic cells, and grade IV indicates total necrosis. Grades I/II are referred to as a poor response, whereas grades III/IV are labelled a good response. However, treatment adjustments based on Huvos grading have not boosted survival rates, leading to its diminished use.^1^, ^8^

Recent advances in biotechnology have enabled the integration of multiomics data from tumour biopsies to refine prognosis predictions and individualize cancer treatments.^9^ For instance, multiomics data was employed to stratify OSA patients into four clusters, pinpointing the *MYC*‐driven cluster with *MYC* amplification, activated mTOR signalling, and dampened immune responses as the most aggressive OSA subtype. This subtype was linked with chemoresistance and the worst outcomes.^10^ However, the high costs of collecting transcriptomic, genomic, and epigenomic profiles could hinder the clinical application of this strategy. Conversely, emerging studies suggest that simplified gene expression signatures could predict OSA outcomes cost‐effectively.^11^, ^12^ However, many of these signatures are based on specific gene sets or small datasets, resulting in doubts regarding their potential for application in clinical settings.

To address these challenges, we employed a methodology encompassing 101 machine‐learning combinations proven effective in autonomously selecting pivotal genes from whole transcriptomic profiles to develop dependable cancer prognostic models.^13^, ^14^ Utilizing this artificial intelligence‐driven approach, we developed a prognostic model based on combined algorithms of CoxBoost and gradient boosting machine (GBM) to predict overall survival (OS) for OSA patients. From this model, we derived a risk score for each patient, termed the artificial intelligence‐derived prognostic index (AIDPI). This innovative metric identified a high‐risk OSA subset resistant to neoadjuvant chemotherapy with a poor prognosis, characterized by molecular traits such as *MYC* overexpression, upregulated mTORC1 signalling and decreased immune cell infiltration. Notably, these features aligned with the characteristics of the previously reported *MYC*‐driven subtype. However, therapeutic guidance for this OSA subtype is still lacking.^10^

Our study further identified squalene epoxidase (SQLE), a crucial rate‐limiting enzyme for cholesterol biosynthesis,^15^ as a metabolic vulnerability for these high‐risk OSA patients. Silencing *SQLE* showed promise in inhibiting OSA both in vitro and in vivo, mirroring a recent study demonstrating that deploying a fungal SQLE protein inhibitor, terbinafine, at 25 μM curtailed OSA in vitro.^16^ However, this study did not elucidate the underlying mechanisms or explore terbinafine's in vivo anti‐OSA effects, raising questions about the feasibility of achieving such a high concentration in vivo and casting doubt on its translational potential. In contrast, our investigation provides deeper insights, revealing that the anti‐OSA effects of silencing *SQLE* were achieved through depleting cholesterol and subsequently inhibiting the FAK/PI3K/Akt/mTOR signalling pathway. We also introduced FR194738, a specific mammalian SQLE inhibitor, which not only suppressed OSA cell growth at a dose of just 2.5 μM but also demonstrated significant anti‐OSA efficacy in vivo without noticeable side effects. Significantly, FR194738 demonstrated synergistic effects with MAP regimen agents in targeting OSA cells, underscoring its potential as a novel therapeutic for the precision management of high‐risk OSA patients.

## 2 METHODS

### 2.1 Selection of datasets

To develop and validate the AIDPI, we collected public datasets based on the following criteria: (1) Tumours were confirmed as OSA by histology. (2) The datasets had complete OS‐associated information. (3) RNA sequencing (RNA‐seq) or microarray detection was performed on fresh‐frozen biopsy samples.

### 2.2 Datasets from the TARGET programme

We obtained multiomics data, including bulk RNA‐seq data, gene‐level copy number variants, masked somatic mutation profiles, masked DNA methylome intensities, and relevant clinical data from the Therapeutically Applicable Research to Generate Effective Treatments (TARGET) programme via Genomic Data Commons Data Portal (GDC, https://portal.gdc.cancer.gov/) (up to 10, October 2022) using TCGAbiolinks.^17^ Hereafter, this dataset is referred to as TARGET‐OSA (*n* = 85). Gene expression levels were calculated as log2‐transformed transcripts per kilobase million (TPM) with a pseudocount value of one. The average RNA expression value was used when duplicate data were found.

### 2.3 Datasets from the GEO

We sourced various datasets from the Gene Expression Omnibus (GEO) via GEOquery, including GSE21257 (*n* = 53),^18^ GSE33382 (*n* = 82),^19^ GSE16091 (*n* = 34),^20^ GSE14827 (*n* = 27),^21^ GSE87437 (*n* = 21),^22^ GSE42352 (3 osteoblasts [OBs], 12 mesenchymal stem cells [MSCs] and 19 OSA cell lines),^19^ GSE16089 (MTX‐resistant Saos2 and its parent cell line with 3 replicates, respectively),^23^ GSE9967 (RNA‐seq data of OSA samples and paired normal bone tissue from 18 patients)^24^ and GSE238110 (RNA‐seq data of 186 primary canine OSA samples).^25^ The survival information of GSE33382 was obtained from R2: Genomics Analysis and Visualization Platform (http://r2.amc.nl). The survival data of GSE238110 were obtained from the Supporting Information of the corresponding article.^25^ We employed the oligo package and the beadarray package to process the raw data from Affymetrix arrays and Illumina BeadChip, respectively.^26^, ^27^ Microarray data annotation was achieved by using relevant packages (Table S2). All probes were initially annotated into Ensembl IDs and subsequently converted to official symbols, following the annotation file of the TARGET‐OSA dataset. For RNA‐seq datasets, the downloaded read count data were converted to TPM using the IOBR package^28^ and underwent log2‐transformation with a pseudocount value of one. The average RNA expression value was taken when duplicate data were found.

### 2.4 Integration of multiple datasets

We merged GSE21257 and GSE16091 into the GEO‐OSA cohort. Moreover, a meta‐OSA cohort was constructed by integrating GSE21257, GSE16091, GSE33382 and TARGET‐OSA. Another combined cohort, OSA‐Huvos, consists of biopsy samples with information on Huvos grade from GSE21257, GSE33382, GSE14827, GSE87437 and TARGET‐OSA. When performing integration, we only included the genes detected across all datasets. The Rank‐In algorithm was employed to mitigate batch effects during dataset integration, and input files, including expression data, sample class and platform information, were prepared according to the corresponding example data (http://www.badd‐cao.net/rank‐in/submission.html).^29^ Principal component analysis (PCA) was conducted using FactoMineR and visualized using the factoextra package.

### 2.5 Datasets from the Cancer Cell Line Encyclopedia (CCLE) project

For cell lines’ multiomics data from Cancer Cell Line Encyclopedia (CCLE), we directly downloaded bulk RNA‐seq, gene‐level copy number data and model information from the DepMap portal (https://depmap.org/portal/, Public 23Q2). Immunofluorescence images of U2OS cells were sourced from The Human Protein Atlas (https://www.proteinatlas.org/).

### 2.6 Single‐cell and bulk RNA‐seq datasets from the sequence read archive (SRA)

We retrieved raw data from single‐cell RNA sequencing (scRNA‐seq) of six OSA biopsy samples (PRJNA681896)^30^ using sra‐tools (https://github.com/ncbi/sra‐tools) and analysed them with Cell Ranger (version 7.1) to generate expression profiles. Seurat was used for further data processing and visualization.^31^ We filtered out cells with fewer than 300 expressed genes or with over 10% mitochondrial genes. Potential doublets were removed using DoubletFinder.^32^ Batch effects were mitigated using Harmony.^33^ Furthermore, cell‐type annotations were achieved by using the scGate and infercna packages.^34^, ^35^ Differentially expressed genes (DEGs) for each of the annotated cell clusters were obtained using the FindAllmarkers function in Seurat with default settings.

To obtain the raw data of the PRJNA698672 BioProject (including OSA tissues and paired adjacent normal tissues from four patients), we used sra‐tools. We obtained reference genome files (hg38) and annotation files from the GDC. We employed the latest TCGA mRNA analysis pipeline (Dr32) for sample analysis and integration. Detailed methodologies are available on the official GDC website.

### 2.7 Pharmacogenomic datasets

The PharmacoGx package was used to download and analyse pharmacogenomic datasets, including GDSC_2020 (v1‐8.2) and GDSC_2020 (v2‐8.2).^36^ The DrugSensitivitySig function within this package was used to analyse the association between SQLE mRNA expression and the area above the dose–response curve (AAC) in OSA cell lines.

### 2.8 Construction of the AIDPI

The AIDPI model for OSA was created following a well‐established workflow,^13^, ^14^ which integrated ten classical machine‐learning algorithms, including least absolute shrinkage and selection operator (LASSO), GBM, random forest (RSF), partial least squares regression for Cox (plsRcox), stepwise Cox (StepCox), supervised principal components (SuperPC), ridge, survival support vector machine (Survival‐SVM), CoxBoost and elastic network (Enet). Among them, RSF, LASSO, CoxBoost, StepCox‐both directions, and StepCox‐backward direction were employed for the first‐step dimensionality reduction and variable screening, which were combined with other algorithms, resulting in 101 machine‐learning algorithm combinations. The generation of the AIDPI involved the following steps: (1) We performed univariate Cox regression analysis using the coxph function of the survival package on both training and validation sets. Consistent prognostic genes (CPGs) were identified based on the criteria of *p*‐values below .01 and consistent hazard ratios of either over one or below one in both cohorts. (2) We utilized the 101 combinations to select genes from the CPGs and fit multiple prognostic models in the training set based on the *Z* score of these genes’ expression values. (3) All models were assessed by calculating risk scores for patients in the training set, validation set and an independent test set using the predict function from the corresponding package based on established models. (4) Harrell's concordance index (C‐index) was calculated by performing univariate Cox regression analysis for the risk scores of all models across these three sets. (5) The model displaying the highest average C‐index was automatically selected as optimal. The risk score calculated based on this optimal model was termed the AIDPI.

### 2.9 Evaluating the predictive value of the AIDPI

We assessed the predictive value of the AIDPI across multiple cohorts, including a training set (GEO‐OSA), validation set (TARGET‐OSA), independent test set (GSE33382‐OSA), GSE21257‐OSA, GSE16091‐OSA and meta‐OSA, via time‐dependent receiver operating characteristic curve (tROC) analysis, performed using timeROC. To determine the optimal threshold for AIDPI in the training set, we used the surv_cutpoint function from the survminer package. Based on this identified threshold, patients in each cohort were categorized into either a low‐ or high‐AIDPI group. Kaplan–Meier survival analysis (KMSA) was performed to delineate the survival differences between these two groups, utilizing the survival package for analysis and the survminer package for visualization.

### 2.10 Comparison of published OSA signatures

We reviewed PubMed for articles on prognostic signatures of OSA published up to May 28th, 2023. Subsequently, we calculated risk scores in all of the mentioned OSA cohorts based on the *Z* scores of gene expression values and the coefficients provided by the articles (Table S1). The predictive performance of these models was evaluated using univariate Cox regression analysis. We employed the compareC package to assess the statistical significance of differences in the C‐index between two distinct signatures.^37^

### 2.11 Construction of integrated models for optimized risk stratification

In the meta‐OSA cohort, we performed univariate Cox regression and visualized results using the show_forest function of the ezcox package. The ezcox_group function was employed to perform univariate Cox regression analysis in subgroups. Multivariate Cox regression analysis was carried out using the coxph function from the survival package, and the results were displayed with the forestmodel package. To generate a nomogram for prediction, we used the regplot package. The calibration curve was created using the rms package, and decision curve analysis (DCA) was conducted using ggDCA. The pROC generated smooth ROC curves. Finally, stacked column charts were created using the ggstatplot package. To enhance the user experience with our AIDPI‐based nomogram, we developed a dedicated application employing the Shiny package. This application facilitates the calculation of the AIDPI. Furthermore, we used the DynNom package to design and deploy a dynamic, interactive nomogram on a web server.

### 2.12 Identification of AIDPI‐related biological processes

To identify biological processes related to AIDPI, we performed differential expression analysis (DEA) using DESeq2. For gene set enrichment analysis (GSEA), we used the clusterProfiler package and gene sets provided by the msigdbr package. Genes with adjusted *p*‐values less than .05 and |log2FoldChange| > mean (|log2FoldChange|) + 2 SD (|log2FoldChange|) were considered DEGs, which were then used for KEGG pathway enrichment analysis. A heat map was generated using ComplexHeatmap.^38^ To analyse masked somatic mutation data, we used the maftools package.^39^ We employed ChAMP to analyse the masked intensity data of the DNA methylome data.^40^ IOBR was used to evaluate the immune score based on expression data.^28^ EpiDISH was used to infer the fraction of infiltrated immune and stromal cells based on methylome data.^41^

### 2.13 Cells and cell culture

OSA cell lines, including U2OS, MNNG/HOS (MNNG), 143B, Saos2, KHOS/NP (KHOS) and MG63, were obtained from the Cell Bank of the Chinese Academy of Sciences (Shanghai, China) and authenticated via short tandem repeat profiling. Additionally, cultivation procedures for all cell lines strictly adhered to the guidelines provided by the cell bank.

### 2.14 Plasmid construction

To modulate gene expression, we designed short hairpin RNA (shRNA) constructs to target SQLE mRNA (shSQLE), along with nontargeting scramble sequences (shControl). These constructs were cloned and inserted into the pGIPZ plasmid (Addgene). The specific targeting sequences for the shRNAs can be found in Table S3. Verification of all constructs was carried out through Sanger sequencing.

### 2.15 Lentivirus production

HEK293T cells were cultured in 10‐cm dishes at 1 × 10^7^ cells per dish. Transfection was performed using Lipofectamine 3000 (Thermo Fisher Scientific) along with the respective plasmids and packaging plasmid for lentiviral constructs (Youbio) according to their protocol. The medium was replaced 6 h posttransfection, and the viral supernatants were harvested after 48 h for subsequent infection of OSA cell lines.

### 2.16 Selection of stably transfected cell lines

Stably transfected cell lines were established through a 2‐week selection process utilizing puromycin at 2 μg/mL (Thermo Fisher Scientific). Gene knockdown or overexpression efficacy was verified via quantitative real‐time PCR (qRT‐PCR) or Western blotting.

### 2.17 RNA extraction and qRT‐PCR

Total RNA was extracted using TRIzol Reagent (Thermo Fisher Scientific) and converted into cDNA using the TransScript All‐in‐One First‐Strand cDNA Synthesis SuperMix for qPCR kit (TransGen Biotech). The qRT‐PCR assays were performed using QuantStudio 5 (Thermo Fisher Scientific) and 2×RealStar Fast SYBR qPCR Mix (GenStar). Experimental procedures followed the manufacturers’ guidelines, and primer sequences are provided in Table S4.

### 2.18 Western blotting analysis

Whole‐cell proteins were extracted using RIPA lysis buffer supplemented with protease and phosphatase inhibitor cocktails (Thermo Fisher Scientific). Equal amounts of cell homogenates were separated by sodium dodecyl sulphate–polyacrylamide gel electrophoresis and transferred onto .45‐μm polyvinylidene difluoride membranes (Millipore), which were blocked with 5% bovine serum albumin in TBST for phosphorylated proteins or 5% nonfat milk in TBST for nonphosphorylated proteins. Primary antibodies were incubated overnight at 4°C, followed by incubation with horseradish peroxidase‐linked secondary antibodies. Protein bands were visualized using SuperSignal West Pico reagents (Thermo Fisher Scientific) and quantified using ImageJ software (version 1.53a). Antibody details are provided in Table S5.

### 2.19 Drug and cholesterol preparation

FR194738 (MCE, Cat HY‐100303), terbinafine (MCE, Cat HY‐17395A), naftifine (Selleck, Cat S3156), MTX (Selleck, Cat S1210), ADM (MCE, Cat HY‐15142A), DDP (MCE, Cat HY‐17394) and cholesterol (Solarbio, Cat C8280) were prepared as stock solutions according to their respective instructions and subsequently diluted to various working concentrations.

### 2.20 Cell proliferation assay

Cells were seeded in quintuplicate in 96‐well plates at 2000 cells per well. Cell viability was assessed at various time points. After removing the old medium, 100 μL of fresh culture medium containing 10% CCK‐8 reagent (Sangon Biotech) was added to each well and incubated at 37°C for 30 min. The optical density (OD) at 450 nm was measured using a microplate reader (BioTek), with each OD value normalized to average at the initial time point to obtain relative cell viability.

### 2.21 Drug sensitivity assay

Cells were seeded in three or more replicates into 96‐well plates at a density of 5000 cells per well and subsequently exposed to varying concentrations of drugs for at least 48 h. The CCK‐8 assay was then performed as described previously. In several experiments, cell viability was determined when adding drugs to calculate the GR_50_, which indicates the concentration of the drug at which the cell growth rate (GR) is half that of the control group. This new metric corrects for confounders in measuring sensitivity to cancer drugs, such as natural differences in the proliferation rates of different cancer cells.^42^ The GRmetrics package was employed to analyse dose–response data and perform visualization.^43^

### 2.22 Synergistic effect assay

Cells were seeded in quadruplicate in 96‐well plates at a density of 5000 cells per well and treated with various drug combination concentrations for 48 h. Subsequently, the CCK‐8 assay was conducted as described earlier. The package SynergyFinder in R software (version 4.3.0) was used to calculate and visualize synergy scores according to four major synergy scoring models, including the Highest Single Agent, Loewe Additivity (Loewe), Bliss Independence (Bliss) and Zero Interaction Potency models.^44^

### 2.23 Colony formation assay

Cells were seeded at a density of 1000 cells per well in 6‐cm dishes (NEST) and cultured for 14 days, with medium changes every 5 days. Cells were fixed with ice‐cold methanol and stained with a crystal violet solution (Beyotime). Colonies consisting of more than 50 cells were counted.

### 2.24 Transwell migration and Matrigel invasion assays

For transwell assay, cells were pretreated with 10 μg/mL mitomycin C (Selleck) for 2 h, then a total of 5 × 10^4^ cells in 100 μL of serum‐free medium were seeded into the upper Transwell chambers, and 600 μL of 10% FBS‐containing medium was added to the lower chambers. After a 24‐h incubation period, cells on the upper side of the Transwell membrane were removed with cotton swabs, and the cells that passed through the membrane to the lower chamber were fixed with 4% paraformaldehyde, stained with 1% crystal violet and photographed. The absorbance at 590 nm was measured following crystal violet elution with 33% glacial acetic acid.

For Matrigel invasion assays, 2 × 10^5^ cells were seeded into Corning BioCoat Matrigel Invasion Chambers. Cells were then processed and analysed similarly to the migration assay.

### 2.25 Apoptosis assay

Apoptosis was assessed using Annexin V‐APC/7‐AAD staining, following the manufacturer's instructions (YEASEN). Flow cytometry was performed using an Attune NxT flow cytometer (Thermo Fisher Scientific). Flow cytometry data were imported and analysed using the flowCore and flowGate packages, with visualization facilitated by the ggcyto package in R software (version 4.3.0).

### 2.26 RNA‐seq and data analysis

High‐throughput mRNA sequencing was conducted by NewCore Biotech. In brief, total RNA was extracted using TRIzol Reagent (Thermo Fisher Scientific), and mRNA purification was performed using the NEBNext Poly(A) mRNA Magnetic Isolation Module Kit (NEB) following the provided manual. According to the manufacturer's instructions, mRNA libraries were prepared using an Illumina TrueSeq mRNA sample preparation kit (Cat RS‐122‐2101). Sequencing was performed on an Illumina NovaSeq 6000 instrument, generating 151 bp paired‐end reads. NewCore BioTech also conducted bioinformatics data analysis. Fastp software (version 0.20.0) was used to trim adapters and remove low‐quality reads, resulting in high‐quality and clean reads. These clean reads were aligned to the human reference genome (hg38) using STAR software (version 2.7.9a). FeatureCounts software (version 2.0) was employed to obtain raw gene‐level mRNA read counts. DEA was conducted as previously described.

### 2.27 Measurement of cholesterol content

Cell and tissue homogenates were prepared for cholesterol extraction using ethanol. The cholesterol content was quantitatively measured using a commercial kit (Jiancheng).

### 2.28 Xenograft models

Female BALB/c nude mice, aged 4–5 weeks, were procured from Hangzhou Ziyuan Experimental Animal Technology Co. Ltd. Mice were housed in cages with a maximum of five mice per cage.

To assess subcutaneous tumourigenic potential, we injected 2 × 10^6^ U2OS cells stably expressing shSQLE or shControl in .1 mL of PBS into nude mice (*n* = 5 per group). Tumour growth was monitored every 3 days, and tumour volume was calculated using the formula: volume = .5 × length × width^2^. After 26 days, the mice were euthanized for further analyses, including tumour photography, weighing, immunohistochemistry (IHC) assay, and detection of cholesterol levels.

To assess the efficacy and safety of FR194738, MNNG cells in .1 mL of Matrigel (Corning, Cat 354234) were subcutaneously injected into nude mice (5 × 10^6^ cells per mouse). Tumour growth and body weight were monitored every 3 days. After 9 days, when the tumour volume reached an average of approximately 60 mm^3^, the mice were divided into two groups (*n* = 5 per group). One group received daily intraperitoneal injection of 10% DMSO, whereas the other group received 100 mg/kg FR194738. Sixteen days after cell injection, the mice were euthanized for further analysis, as mentioned earlier. The tumour growth inhibition (TGI) rate was calculated based on the average tumour weights using the formula: TGI = [1 − (FR194738 group/vehicle group)] × 100.

### 2.29 Tissue microarray (TMA) and immunohistochemistry (IHC)

From YEPCOME Biotechnology, we purchased a TMA (Cat YP‐BonSur2201) (*n* = 79) to analyse the association of SQLE protein expression with the prognoses of OSA patients. In this TMA, two samples were excluded because no sarcoma cells were found in them. Therefore, only 77 samples were analysed further. IHC staining was performed using standard procedures with SQLE antibody (H‐6) (Santa Cruz Biotechnology, Cat sc‐271651). All slides were scanned with a Pannoramic Midi II digital slide scanner (3DHISTECH Ltd.). The demographic characteristics and clinical data of the patients from the TMA are listed in Table S6.

All tumour tissues from mice were fixed in 4% paraformaldehyde for subsequent paraffin embedding and cut into 5 μm thick sections. IHC staining was performed using standard procedures with SQLE antibody (Proteintech, Cat 12544‐1‐AP), Ki‐67 antibody (Abcam, Cat ab15580), and cleaved caspase‐3 (Asp175) antibody (Cell Signaling Technology, Cat 9661). The details of the antibodies used are listed in Table S5.

The staining results were analysed with HALO image analysis software (Indica Labs). The average OD was defined as the integrated OD divided by the area of the region of interest (ROI) and used to represent the strength of SQLE abundance.

### 2.30 Statistical and computational analyses

All results were derived from at least three independent experiments, and data from one representative experiment are shown. The sample size for all in vitro and in vivo experiments was chosen empirically.

Unless stated otherwise, all statistical analyses were conducted using R software (version 4.2.3). Details of the main packages and web tools used in this study are displayed in Table S7. We used the shapiro_test function from the rstatix package to assess normality. For correlation analyses, the Pearson correlation coefficient was calculated when both variables met the assumptions of normality. Otherwise, Spearman's method was used. Levene's test was applied using the levene_test function in the rstatix package to assess the homogeneity of variance across groups. Parametric statistical tests were used when the data met the assumptions of both normality and homogeneity of variances. Otherwise, nonparametric tests were employed. Unless specified otherwise, all experiments used at least three technical replicates in an independent test. If parametric statistical tests could be used, data were presented as bar‐dot plots, with horizontal bars representing means and whiskers indicating standard deviations (SD). Otherwise, boxplots were used to present the data. For two independent groups, comparisons were conducted using either Student's *t*‐test (parametric) or Wilcoxon rank‐sum test (nonparametric). Either one‐way ANOVA (parametric) or the Kruskal–Wallis rank sum test (nonparametric) was used to compare multiple groups. *p‐*Value adjustments for multiple comparisons were performed using Holm's method.

For cell proliferation and tumour growth curve analyses, repeated‐measures ANOVA with the Greenhouse–Geisser correction and Tukey's honestly significant difference test were performed using the anova_test function and the Tukey_hsd functions in the rstatix package. Unless otherwise mentioned, all *p*‐values were derived from two‐sided statistical tests, and a *p*‐value or adjusted *p*‐value less than .05 was considered to indicate statistical significance.

## 3 RESULTS

### 3.1 Development and validation of the AIDPI

**FIGURE 1:**
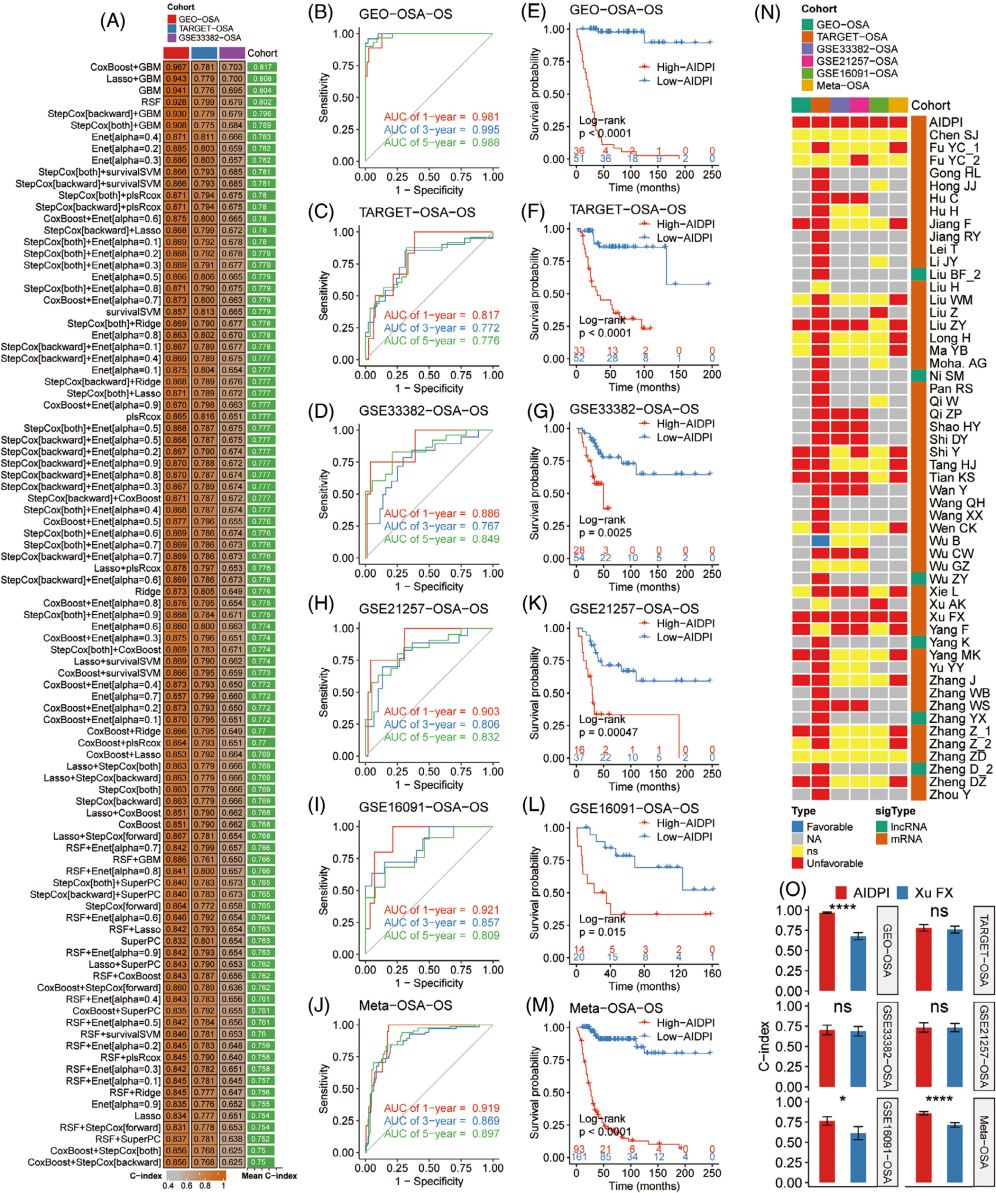
Development and validation of the artificial intelligence‐derived prognostic index (AIDPI). (A) C‐indices of multiple models derived from various machine‐learning algorithm combinations in three cohorts. (B–D and H–J) Time‐dependent ROC curve analysis of the AIDPI across multiple cohorts. (E–G and K–M) Kaplan–Meier survival analyses of the AIDPI across multiple cohorts. (N) Results of univariate Cox regression analyses of the AIDPI and previously published prognostic signatures. (O) Comparison of the C‐indices of the AIDPI and a signature established by Xu et al., ns: *p* > .05; *p < .05; ****p < .0001 by *Z* test.

We developed the AIDPI following the schematic in Figure S1A. Initially, we merged two cohorts, GSE21257‐OSA and GSE16091‐OSA, into the GEO‐OSA cohort and mitigated batch effects (Figure S1B), and this cohort was employed as a training set. Meanwhile, TARGET‐OSA served as our validation set. We identified 18 CPGs shared between the training and validation sets (Figure S1C). These CPGs were input into a machine‐learning framework to generate multiple prognostic models in the training set. After evaluation in the training set, validation set and an independent test set (GSE33382‐OSA), the model established by the combination of CoxBoost and GBM was chosen as the optimal model as its average C‐index (.817) was the highest one (Figure 1A). This model comprised 12 genes, which are presented with their relative influences in Figure S1D. Utilizing this model, we calculated the AIDPI for every patient across multiple cohorts. The tROC analyses revealed that the areas under the ROC curves (AUCs) in the training set were .981, .995 and .988 for 1‐, 3‐ and 5‐year OS, respectively (Figure 1B). The AUCs in the validation set were .817, .772 and .776 (Figure 1C). For the independent test set, the AUCs were .886, .767 and .849 (Figure 1D). Based on the optimal threshold of the AIDPI determined from the training set (Figure S1E), OSA patients were categorized into low‐ and high‐AIDPI groups. KMSA revealed notably adverse outcomes for the high‐AIDPI group in the training set (*p* < .0001) (Figure 1E), validation set (*p* < .0001) (Figure 1F) and independent test set (*p* = .0025) (Figure 1G). This trend was consistently observed across other cohorts (Figure 1H–M), including a combined meta‐OSA cohort (Figure 1J,M). The harmonization in the transcriptomic profile after removing the batch effect, coupled with congruent OS results across the four datasets, justified their consolidation into a singular meta‐OSA cohort (Figure S1F,G).

We further assessed the predictive ability of the AIDPI and 68 previously published OSA signatures (Table S1) in the six mentioned cohorts. However, because of gene naming alterations and gene absences in microarray datasets, only 53 published signatures and the AIDPI were tested in at least one cohort, and the results were visualized in a heat map (Figure 1N). Strikingly, this heat map showed that only two signatures consistently exhibited statistical significance across all cohorts: our AIDPI and Xu et al.’s signature.^12^ A direct C‐index comparison underscored AIDPI's definitive superiority over Xu et al.’s signature within three cohorts (Figure 1O). These findings demonstrate that the AIDPI can predict OSA patients’ outcomes and outperforms previously established signatures. Additionally, we deployed an application (https://yongjiewangosa.shinyapps.io/AIDPIshinyAPP/) that can calculate the AIDPI.

### 3.2 Survival prediction enhancement based on the AIDPI and clinical features

**FIGURE 2:**
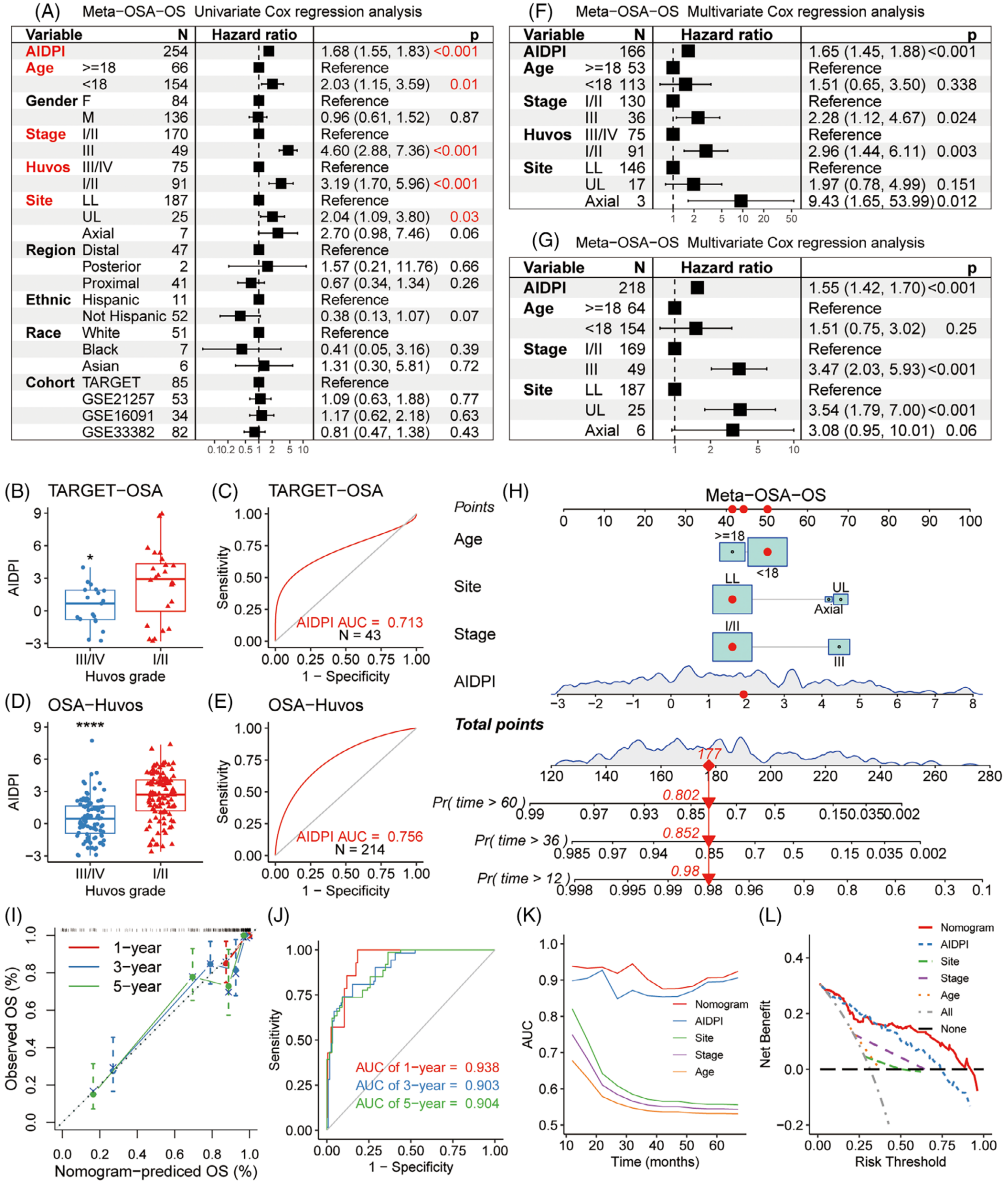
Survival prediction based on artificial intelligence‐derived prognostic index (AIDPI) and clinical features: (A) univariate Cox regression analyses of relations between AIDPI and clinicopathological features regarding prognostic value, (B and D) Boxplots displaying AIDPI distributions across varying Huvos grades in the indicated cohorts, (C and E) the ROC curves for evaluating the ability of the AIDPI to predict the response to neoadjuvant chemotherapy, (F–G) multivariate Cox regression analyses with or without the integration of Huvos grade, (H) a nomogram was derived from meta‐osteosarcoma (OSA), (I) calibration curve of the nomogram, (J) time‐dependent ROC curve analyses of the established nomogram, (K) the predictive performances were compared among various factors, (L) decision curve analysis underscores the superior net benefit of the nomogram relative to other indicators. *p < .05; ****p < .0001 by Wilcoxon rank‐sum test (B and D).

Given the recognized impact of multiple clinical variables on OSA patient outcomes, we sought to elucidate relationships between these factors and the AIDPI for authenticating the AIDPI as an independent prognostic biomarker and enabling a comprehensive model for enhanced survival prediction. Univariate Cox regression analyses in meta‐OSA revealed significant associations between patients’ OS and parameters such as AIDPI, age, MSTS stage, Huvos grade and primary tumour site (Figure 2A). Further analysis of these variables showed that patients with MSTS stage I/II, Huvos grade III/IV, age over 18 years old or primary tumours in the lower limbs tended to exhibit low AIDPI values (Figure S2A–D). Furthermore, the AIDPI consistently emerged as an unfavourable prognostic indicator across varying patient subgroups determined by these clinical factors, with the exception of those with axial skeleton tumours, potentially due to the small sample size of this subset (Figure S2E–H).

In the TARGET‐OSA cohort, where Huvos grade was available for 43 patients, we observed a clear trend that a high AIDPI score corresponded to Huvos grade I/II (Figure 2B). When we assessed its predictive power in chemotherapy responses, the AIDPI yielded an AUC of .713 (Figure 2C). This predictive property was reaffirmed in an expanded OSA‐Huvos dataset consisting of samples with information on Huvos grade from five datasets (Figure S2I), where once again Huvos grade I/II corresponded to elevated AIDPI score (Figure 2D), and AIDPI achieved an AUC of .756 in predicting the response to neoadjuvant chemotherapy (Figure 2E).

Multivariate Cox regression analysis of the meta‐OSA cohort identified AIDPI, MSTS stage, Huvos grade, and primary tumour site as independent prognostic factors (Figure 2F). Given the Huvos grade with over 25% missing values and the AIDPI's high accuracy in predicting neoadjuvant chemotherapy response, Huvos grade was excluded from the multivariate Cox regression analysis (Figure 2G). Based on this revised model, we constructed a nomogram to predict patients’ survival probability (Figure 2H). Calibration curves (Figure 2I) and tROC evaluations (Figure 2J) confirmed the robust predictive capability of this nomogram, which had AUCs of .938, .903 and .904 for 1‐, 3‐ and 5‐year OS, respectively. Furthermore, this nomogram surpassed other factors in terms of performance based on the AUC analysis (Figure 2K), and DCA revealed that its net benefit was broader than those of other clinical parameters (Figure 2L).

These findings highlight the AIDPI as an independent prognostic indicator. Moreover, the nomogram based on the AIDPI, age, MSTS stage, and primary tumour site has emerged as a tool for prognosis prediction for OSA patients that is superior to isolated clinicopathological features. Additionally, we deployed an application (https://yongjiewangosa.shinyapps.io/AIDPIbasedNomogramForOSA/) that allows real‐time survival estimates using this nomogram.

### 3.3 Identifying dysregulated pathways in high‐AIDPI patients

**FIGURE 3:**
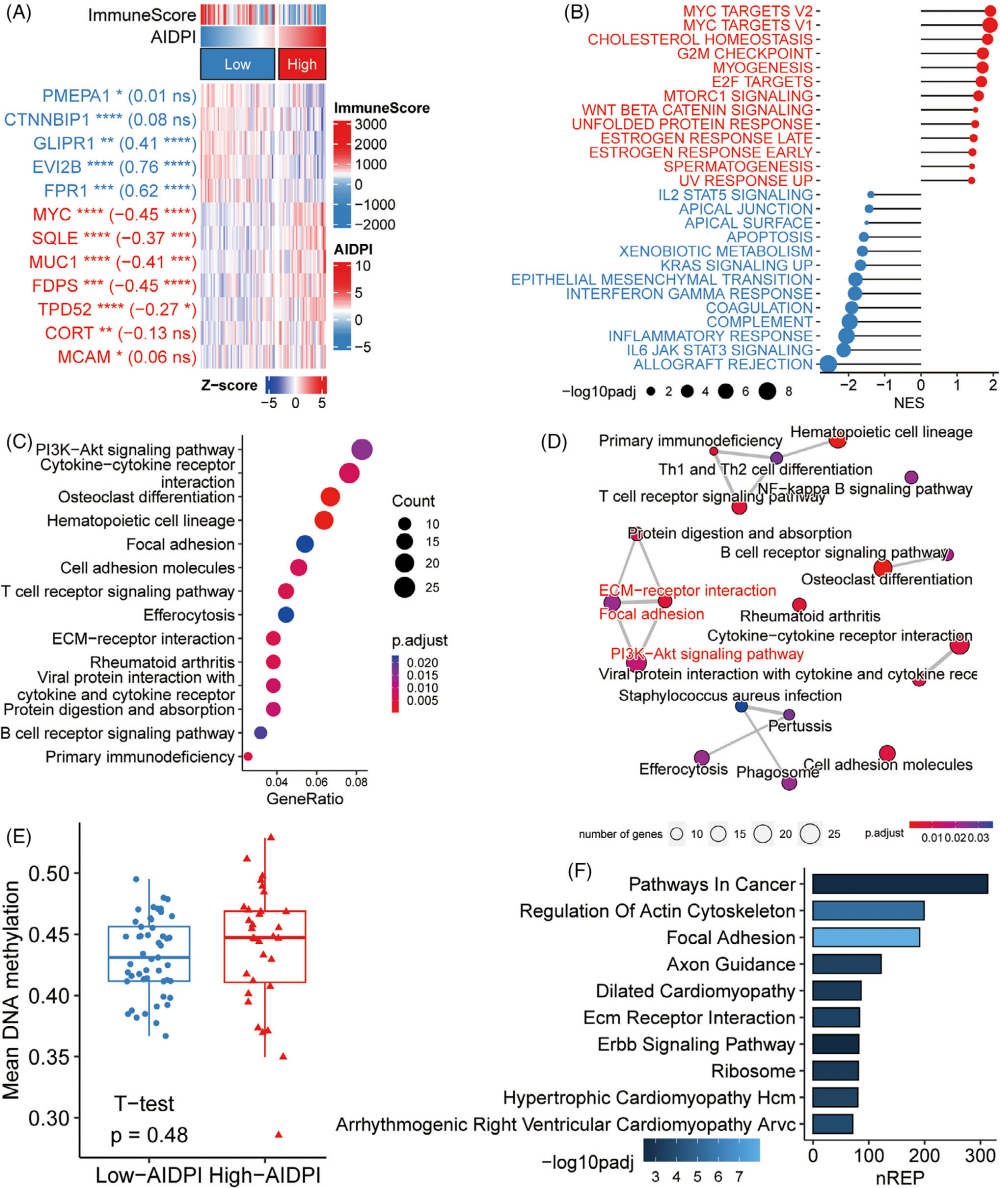
Identifying dysregulated pathways in high‐artificial intelligence‐derived prognostic index (AIDPI) patients. (A) A heat map elucidates the expression of the AIDPI genes and their correlation with the immune score. Stars preceding brackets indicate adjusted *p*‐values from differential expression analysis (DEA), whereas numbers and stars within the brackets convey the correlation coefficient and associated *p*‐value, ns: *p* > .05; *p < .05; **p < .01; ***p < .001; ****p < .0001. (B) Gene set enrichment analysis (GSEA) results spotlight biological processes notably correlated with high (red) or low (blue) AIDPI. (C) A bubble plot shows findings from KEGG enrichment analysis based on differentially expressed genes (DEGs). (D) An enrichment map arranges enriched terms into a network, connecting the terms that have shared genes. (E) Boxplot contrasts average DNA methylation levels between two AIDPI groups. (F) A bar chart manifests empirical Bayes GSEA results based on epigenomic data. nREP, number of genes enriched in this pathway.

A heat map derived from TARGET‐OSA dataset showed the AIDPI, immune score, and expression patterns of the 12 genes used in AIDPI calculations (AIDPI genes). Within the high‐AIDPI group, seven AIDPI genes exhibited marked upregulation, with five being inversely correlated with the immune score. On the other hand, five AIDPI genes manifested notable downregulation, with three displaying a positive correlation with the immune score (Figure 3A). GSEA pinpointed enhanced gene sets in the high‐AIDPI group, including MYC targets V2, MYC targets V1, cholesterol homeostasis and mTORC1 signalling. In contrast, gene sets on apoptosis and specific immune responses were negatively enriched (Figure 3B). KEGG enrichment analysis of the DEGs highlighted pathways vital for OSA progression (Figure 3C), including PI3K–Akt signalling,^45^ cytokine–cytokine receptor interaction,^46^ osteoclast differentiation,^47^ focal adhesion^48^ and extracellular matrix (ECM)‐receptor interaction.^49^ An enrichment map highlighted a significant cluster incorporating the PI3K–Akt signalling pathway, focal adhesion and ECM‐receptor interaction (Figure 3D).

Further exploration aimed to discern the origins of transcriptomic dysregulation by delving into the genomic and epigenomic data. Notably, AIDPI genes lacked somatic mutations. Moreover, there was no significant distinction between the two AIDPI groups in terms of tumour mutation burden (Figure S3A) or mutated genes summarized by pathways (Figure S3B). However, copy number assessments suggested increased copy numbers for the *MCAM*, *MYC* and *SQLE* genes in the high‐AIDPI group (Figure S3C), possibly explaining their overexpression and the dysregulation of corresponding gene sets. Although methylome analysis did not uncover significant shifts in average methylation levels between the two AIDPI groups (Figure 3E), KEGG pathways enriched through empirical Bayes GSEA,^50^ based on epigenomic data revealed the most significant dysregulation in focal adhesion (Figure 3F), mirroring transcriptomic insights (Figure 3C). These findings indicate that the aberrant pathways at the transcriptomic level might be rooted in DNA copy number variations and DNA methylation alterations in the high AIDPI cluster.

Additionally, the transcriptomic analysis highlighted that the hematopoietic cell lineage was enriched (Figure 3C), hinting at immune cell infiltration modifications. Indeed, methylome‐based estimations of cell infiltration revealed a decline in CD4^+^ T cells, monocytes and neutrophils in the high‐AIDPI group, accompanied by an increase in fibroblasts (Figure S3D).

These findings indicate that the unfavourable prognosis of the high‐AIDPI group may stem from pathway alterations triggered by changes in DNA copy numbers, DNA methylation and altered immune cell infiltration patterns in OSA.

### 3.4 Identifying therapeutic targets for high‐AIDPI patients

**FIGURE 4:**
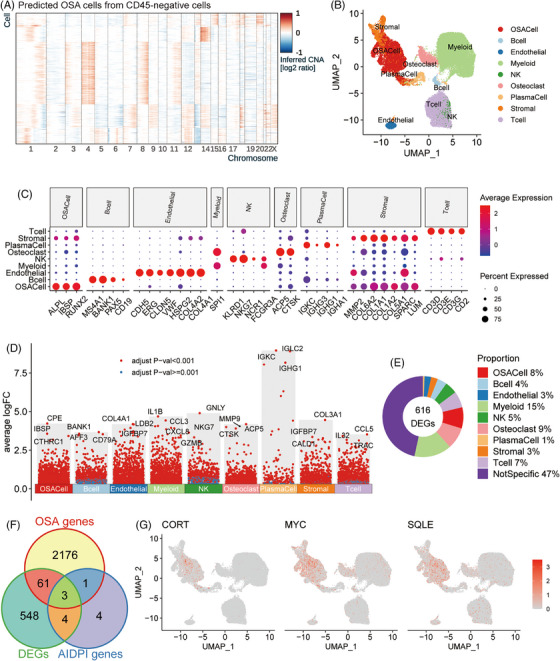
Identifying therapeutic targets for high‐artificial intelligence‐derived prognostic index (AIDPI) patients: (A) A heat map shows inferred CNA in predicted osteosarcoma (OSA) cells, (B) a uniform manifold approximation and projection (UMAP) plot of nine annotated cell types, (C) a bubble plot displays marker gene expression across nine identified cell clusters, (D) in a Manhattan plot, genes highly expressed in specific cell clusters are pinpointed, (E) a donut chart displays the cellular sources of differentially expressed genes (DEGs) between the two AIDPI groups, (F) a Venn diagram presents shared entities among the indicated gene groups and (G) feature plots display normalized expression of the indicated genes across individual cells.

To determine which cell type expressed the DEGs between the low‐ and high‐AIDPI groups from bulk RNA‐seq data and pinpoint therapeutic targets specific to OSA cells, we assessed a scRNA‐seq dataset for six OSA biopsy samples. Utilizing the scGate package,^34^ we identified various cell types in OSA tissues, primarily consisting of stromal and immune cells, including lymphocytes (Figure S4A). We isolated CD45‐positive cells and employed 1000 of them as reference cells for identifying OSA cells. Conversely, CD45‐negative cells were considered potential OSA cell candidates. Using the infercna package,^35^ we inferred copy number alterations (CNAs) based on the average expression of 150 genes per chromosomal region. Relative to the signals of reference cells, those candidates showing heightened CNA signals and robust correlations with the entire cell population were identified as OSA cells, whereas others were classified as normal cells (Figure S4B,C). Distinct chromosomal amplifications and deletions were observed in the predicted OSA cells (Figure 4A) but were absent in both reference and anticipated normal cells (Figure S4D,E). For refined cell annotation, we assessed the mRNA expression levels of selected markers: ACP5 for osteoclasts, VWF for endothelial cells and COL1A1 for stromal cells (Figure S4F,G).^51^, ^52^ The scGate package facilitated automated immune cell annotation, allowing the annotation of nine primary clusters finally, including OSA cells, B cells, endothelial cells, myeloid cells, NK cells, osteoclasts, plasma cells, nontumour stromal cells (stromal) and T cells (Figure 4B). The marker genes defining each cluster were subsequently presented with their expression patterns via a bubble plot (Figure 4C). All delineated cell types were present across the six biopsies, with OSA3 and OSA5 presenting the maximal and minimal OSA cell frequencies, respectively (Figure S4H), consistent with results reported by Liu et al.^30^

Based on the DEA results in the scRNA‐seq dataset, we illustrated positively expressed genes (PEGs) across each cell cluster (Figure 4D), showing that *CPE*, *IBSP* and *CTHRC1* were the top three genes highly expressed in OSA cells. By comparing the DEGs from the low‐ and high‐AIDPI groups with the PEGs of each cell cluster, we discovered that only 8% of DEGs were predominantly expressed in OSA cells (Figure 4E). The intersection of the twelve AIDPI genes with DEGs and PEGs highlighted three common genes (Figure 4F), whose expression patterns were subsequently displayed through feature plots (Figure 4G). According to the canSAR database (https://cansar.ai/),^53^ only the proteins encoded by *MYC* and *SQLE* possess druggable structures and emerge as potential targets for high‐AIDPI patients.

### 3.5 *SQLE* overexpression in OSA correlates with tumour progression

**FIGURE 5:**
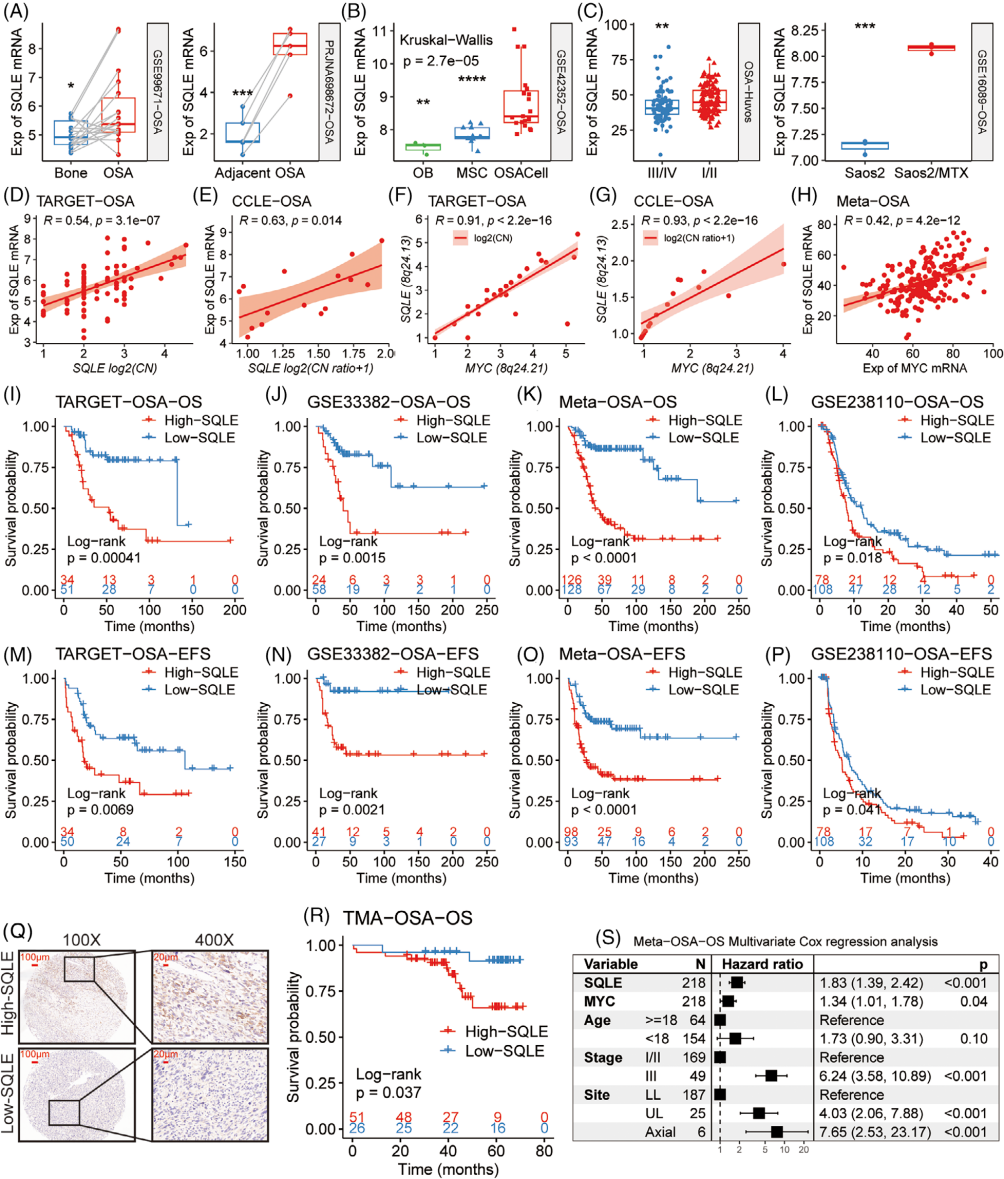
Overexpression of squalene epoxidase (*SQLE*) in osteosarcoma (OSA) correlates with tumour progression. (A–C) Boxplots demonstrate SQLE mRNA levels, contrasting OSA tissues versus their adjacent normal counterparts (A), OSA cell lines (OSACell) versus osteoblasts (OB) and mesenchymal stem cells (MSC) (B), samples with different Huvos grades and methotrexate‐resistant Saos2 (Saos2/MTX) versus its parent cell line (C). (D and E) Scatter plots show the relationships between copy number and mRNA level of *SQLE* in OSA samples and OSA cell lines. (F and G) Correlations between gene‐level copy numbers for MYC and SQLE are portrayed through scatter plots in OSA samples and OSA cell lines. (H) A scatter plot shows the correlation between the mRNA expression of MYC and SQLE in the indicated datasets. (I–P) Kaplan–Meier survival analyses of SQLE mRNA levels were performed across multiple human and canine OSA cohorts. (Q) Representative images of immunohistochemical (IHC) staining of SQLE in the TMA. (R) Kaplan–Meier survival analysis of a tissue microarray (TMA) consisting of 26 OSA patients with low SQLE expression and 51 OSA patients with high SQLE expression. (S) A forest plot shows the results of multivariate Cox regression analysis. CN, copy number; Exp, expression. *p < .05; **p < .01; ***p < .001; ****p < .0001, by Wilcoxon rank‐sum test (A and C) and adjusted using Holm's method (B).

Traditionally, elevated *MYC* expression has been linked to the adverse prognosis of OSA.^54^ Focusing on *SQLE*, we observed a marked increase in its expression in OSA tissues and cellular models when compared to their normal adjacent tissues and putative progenitor cells, including OB and MSCs (Figure 5A,B). Notably, OSA specimens with Huvos grade I/II, indicating poor response to neoadjuvant chemotherapy, manifested elevated *SQLE* expression, and the same trend was also observed in MTX‐resistant Saos2 cells (Saos2/MTX) compared to its parent cell line (Figure 5C). These findings underscore the potential significance of *SQLE* in the initiation and chemoresistance of OSA.

We further investigated potential molecular mechanisms underlying *SQLE* overexpression in OSA. A robust positive correlation was observed between the *SQLE* gene‐level copy number and its mRNA expression level within both OSA tissues and cell lines (Figure 5D,E). The oncogene *MYC*, frequently amplified in OSA,^55^ is proximate to *SQLE* in genomic location (8q24.13 for *SQLE* vs. 8q24.21 for *MYC*). Strong correlations between their gene copy numbers (Figure 5F,G) and mRNA expression levels (Figure 5H) suggest that concurrent *SQLE* and *MYC* amplifications might lead to their increased mRNA levels in OSA.

Elevated *SQLE* expression is also linked to poor outcomes in humans or canines with OSA, as evidenced by KMSA across multiple cohorts, showing that patients in the high‐SQLE group had significantly lower OS or event‐free survival rates across multiple human and canine OSA cohorts (Figure 5I–P). Additionally, we detected the SQLE protein abundance in 77 human OSA tissues using a TMA and displayed representative staining images of low and high SQLE protein expression (Figure 5Q), which confirmed that high SQLE protein expression was associated with poor survival in patients with OSA by KMSA (*p* = .037) (Figure 5R). A multivariate Cox regression analysis in meta‐OSA indicated that both SQLE mRNA expression and MYC mRNA expression are independent indicators of mortality risk in OSA patients, even when adjusted for clinical factors such as age, MSTS stage and primary tumour site (Figure 5S).

Although the MYC protein remains a pivotal OSA target,^10^, ^54^ targeting it poses challenges due to its nuclear localization and the absence of defined protein pockets.^56^ In contrast, the SQLE protein, located in the cytosol, as shown in Figure 5Q and Figure S5A, presents a more accessible target due to its nature as a metabolic enzyme. In CCLE, both the gene‐level copy number and mRNA expression level for *SQLE* are much higher than those in normal cells (Figure S5B,C), suggesting that targeting SQLE protein in vivo may impair OSA cells more effectively while sparing normal cells. Furthermore, an inverse correlation was observed between SQLE mRNA expression and the proportion of infiltrating CD4^+^ T cells in TARGET‐OSA samples (Figure S5D), implying SQLE's influence on the immune landscape of OSA.

These results suggest that the overexpression of *SQLE* in OSA, due to its co‐amplification with *MYC* at the DNA level, could promote OSA progression by promoting the chemoresistance of OSA cells and suppressing the infiltration of anti‐OSA immune cells.

### 3.6 *SQLE* knockdown impedes OSA in vitro and in vivo

**FIGURE 6:**
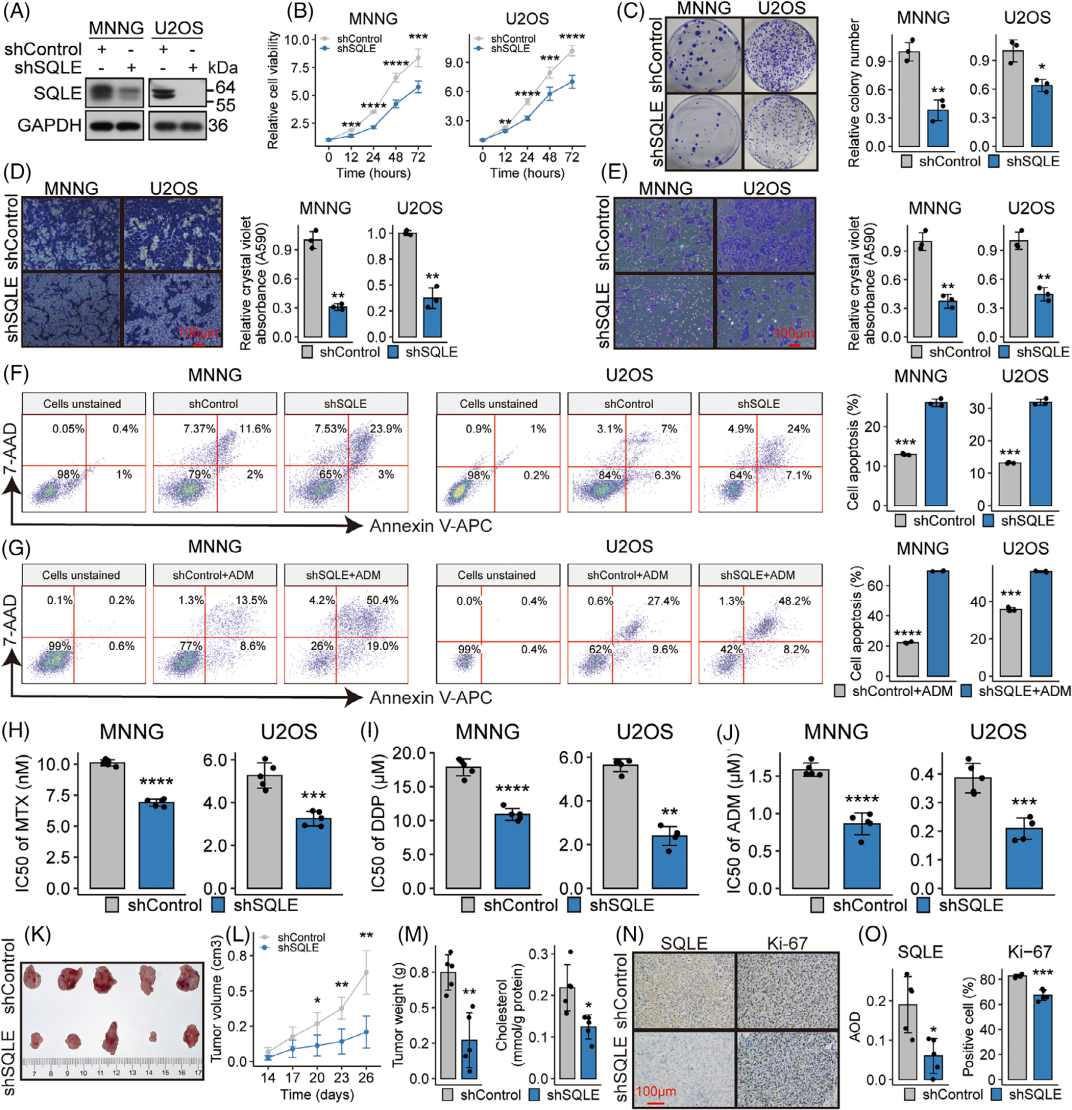
Squalene epoxidase (SQLE) knockdown impedes osteosarcoma (OSA) progression in vitro and in vivo. (A) SQLE protein abundances following transfection of either shSQLE or shControl are depicted via Western blot in labelled cell groups. (B–E) Assays of cellular proliferation (B), clonogenic growth (C), migration (D) and invasion (E) in labelled cell groups. (F and G) Flow cytometric analyses, employing Annexin V‐APC/7‐AAD staining, quantify apoptotic cells in labelled cell groups, either untreated (F) or following adriamycin (ADM) exposure (G). Apoptotic cells exhibit Annexin V‐APC positivity within the right‐top and right‐down quadrants. (H–J) Bar plots contrast the sensitivities of the indicated cell groups to the indicated drugs. (K) Images of isolated tumours from subcutaneous xenograft models established using U2OS cells either harbouring shControl or shSQLE. (L) Tumour growth curves of the indicated groups. (M) Weight and cholesterol levels of tumour masses are presented in bar plots. (N) Representative images of immunohistochemistry for SQLE and Ki‐67 staining in tumours derived from the xenograft model. (O) Bar plots contrast the SQLE abundance and percentage of Ki‐67‐positive cells. AOD: average optical density. *p < .05; **p < .01; ***p < .001; ****p < .0001, by Student's *t*‐test for bar plots, or Tukey HSD test for cellular proliferation and tumour growth curves.

To unravel the role of *SQLE* in OSA, we constructed four shRNAs and selected the optimal one (Figure S6A) to silence *SQLE* in MNNG and U2OS cells (Figure 6A), which have high endogenous SQLE protein expression (Figure S6B). The knockdown of *SQLE* inhibited malignant phenotypes of these cells, including proliferation (Figure 6B), colony formation (Figure 6C), migration (Figure 6D) and invasion (Figure 6E). Furthermore, *SQLE* knockdown promoted apoptosis both in the absence and presence of drug treatment (Figure 6F,G) and enhanced sensitivity to agents from the MAP regimen, as evidenced by decreased IC_50_ values in the shSQLE group (Figure 6H–J and Figure S6C–E). In xenograft models, we found that silencing *SQLE* inhibited tumour growth and decreased the weights and cholesterol levels of tumours (Figure 6K–M). IHC analyses confirmed reduced SQLE protein abundance and Ki‐67‐positive cells post‐*SQLE* silencing (Figure 6N,O). These results demonstrate that targeting the SQLE protein may be a reasonable approach to suppress OSA.

### 3.7 *SQLE* silencing impedes OSA by reducing cholesterol and inhibiting the FAK/PI3K/Akt/mTOR pathway

**FIGURE 7:**
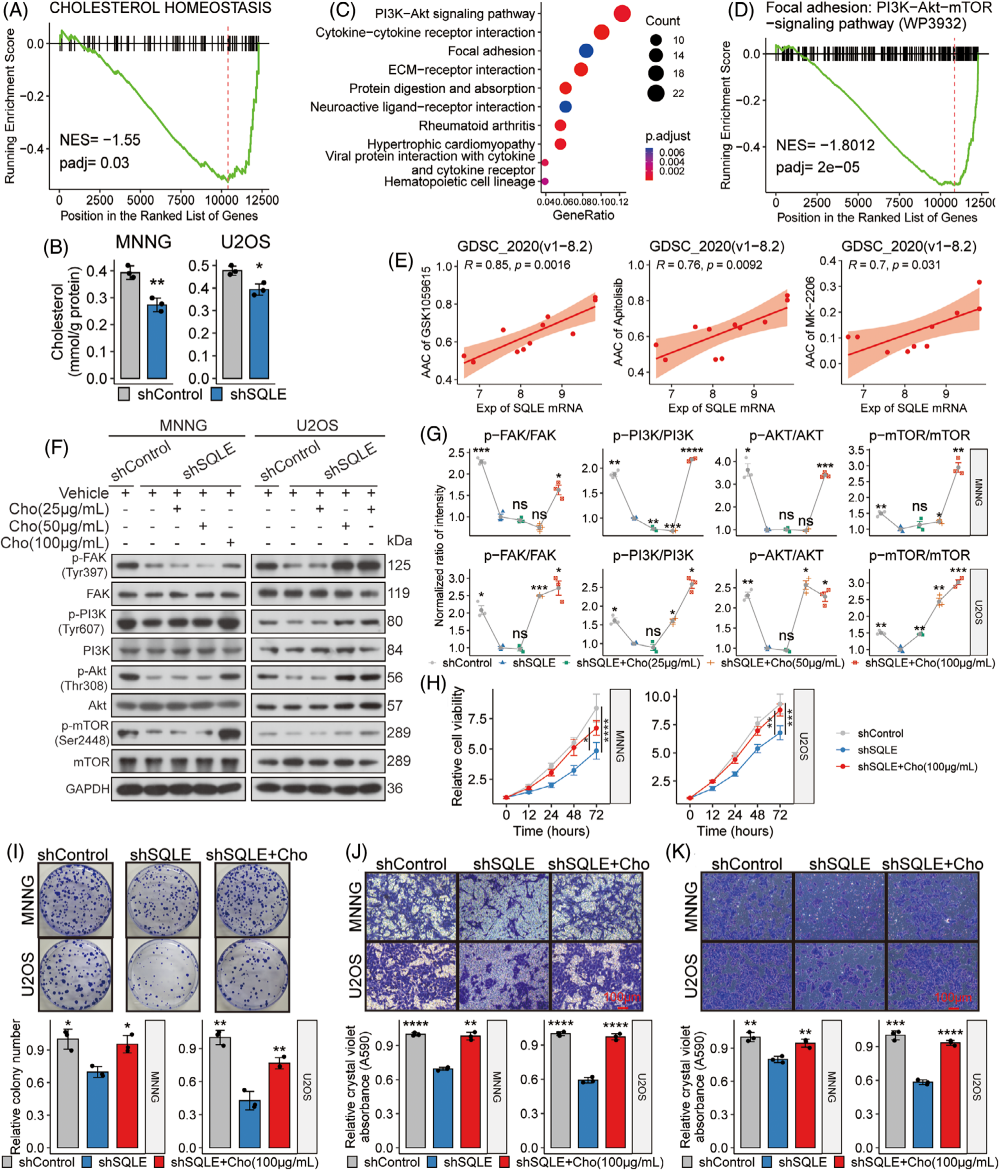
Squalene epoxidase (SQLE) silencing impedes osteosarcoma (OSA) by reducing cholesterol and inhibiting the FAK/PI3K/Akt/mTOR pathway. (A and D) Gene set enrichment analysis (GSEA) plots illustrate enrichment trends for the indicated gene sets. Genes are arranged in descending order based on their log2FoldChange value. (B) The bar graphs show intracellular cholesterol levels in labelled cell groups. (C) KEGG enrichment results of differentially expressed genes (DEGs) are presented in a bubble plot. (E) Scatter plots show correlations between SQLE mRNA levels and area above the dose–response curve (AAC) values of three specific inhibitors targeting the PI3K/mTOR signalling cascade. (F and G) Protein expression profiles in labelled cell groups and subsequent quantifications of these profiles are displayed. (H–K) Experimental outcomes of the indicated cell groups, including cellular proliferation (H), clonogenic potential (I), migration (J) and invasion capabilities (K), are displayed. Cho: Cholesterol. For experiments involving multiple groups, the mean value of the shSQLE group served as a comparative benchmark against other groups. ns: *p* > .05; *p < .05; **p < .01; ***p < .001; ****p < .0001, by Tukey HSD test for all proliferation curves, or Student's *t*‐test adjusted using Holm's method for other plots.

Having discerned the detrimental effect of *SQLE* silencing on OSA, our focus shifted to unveiling the underlying molecular mechanisms. We performed RNA‐seq on U2OS cells that stably expressed either shSQLE or shControl. Differences in transcriptomic profiles between the two groups were displayed by a PCA plot (Figure S7A). A volcano plot displayed an apparent decrease in SQLE mRNA in the shSQLE group (Figure S7B). GSEA revealed a notable downregulation of the cholesterol homeostasis gene set following *SQLE* silencing (Figure 7A), consistent with our findings of reduced intracellular cholesterol in MNNG and U2OS cells after *SQLE* knockdown (Figure 7B). Upon KEGG enrichment of DEGs, the PI3K–Akt signalling pathway was significantly enriched, displaying the highest gene ratio (Figure 7C). An enrichment map was used to integrate enriched terms into a network, suggesting a connection between the PI3K–Akt signalling pathway, focal adhesion and ECM‐receptor interaction (Figure S7C). Indeed, these pathways were combined into a broader gene set, the focal adhesion‐PI3K–Akt‐mTOR pathway (WP3932) in the WikiPathways database, which was similarly downregulated after *SQLE* silencing in U2OS cells (Figure 7D). Further analysis of drug‐gene relationships revealed positive correlations between SQLE mRNA level and AAC values of various agents, notably GSK1059615, an antagonist of the PI3K/mTOR signalling pathway (Figure S7D). Higher AACs suggest increased drug sensitivity due to their inverse relation with the IC_50_ values (Figure S7E). Remarkably, the SQLE mRNA level exhibited a robust correlation with AAC values of three PI3K/mTOR pathway antagonists (Figure 7E), emphasizing possible associations between SQLE mRNA and the activation of this pathway in OSA cell lines.

Considering the role of SQLE protein in cholesterol synthesis and cholesterol's pivotal role in cellular signalling modulation via lipid rafts,^57^ we hypothesized that *SQLE* silencing reduced intracellular cholesterol levels and subsequently deactivated the FAK/PI3K/Akt/mTOR signalling pathway and finally inhibited OSA progression. Consistent with this hypothesis, *SQLE* silencing in MNNG and U2OS cells led to decreased phosphorylation of crucial proteins, including focal adhesion kinase (FAK), PI3K, Akt and mTOR. However, cholesterol supplementation (100 μg/mL) restored these phosphorylation patterns (Figure 7F,G) and counteracted the suppressive effects of *SQLE* knockdown on these OSA cell malignant phenotypes (Figure 7H–K). These findings suggest that *SQLE* silencing inhibits OSA by reducing cholesterol levels and subsequently inhibiting the FAK/PI3K/Akt/mTOR pathway.

### 3.8 Pharmacologic SQLE inhibition suppresses OSA progression and enhances chemotherapy efficacy

The observed suppressive effects of *SQLE* silencing on OSA led us to explore its therapeutic potential further. Fungal SQLE inhibitors such as naftifine and terbinafine have demonstrated anti‐tumour effects on various cancer lines, and they exhibited no effect on healthy fibroblasts at similar concentrations.^16^, ^58^, ^59^ Given their design for fungal SQLE, their utility in human OSA therapy may be constrained. FR194738, however, has been recognized as a potent mammalian SQLE inhibitor, showing superior efficacy and bioavailability compared to its precursor compound.^60^ Remarkably, preclinical studies have confirmed the effectiveness of FR194738 against prostate cancer.^61^ Both MNNG and U2OS cells notably displayed enhanced sensitivity to FR194738, with IC_50_ values markedly lower than those of naftifine and terbinafine (Figure S8A–C), driving us to assess FR194738's therapeutic promise for OSA.

In our initial assessment, we gauged the sensitivity of four distinct OSA cell lines to FR194738. Both the IC_50_ and GR_50_ values for MNNG and U2OS were appreciably reduced in comparison to those of MG63 and 143B cells (Figure S8D–F). At a 72‐h exposure to 4 μM FR194738, a significant decrease in GR and cell viability was observed in MNNG and U2OS cells but not in MG63 and 143B cells (Figure S8G), which might be attributed to the diminished SQLE protein levels in MG63 and 143B cells (Figure S6B), signifying FR194738's specificity for cells expressing SQLE protein. Intriguingly, under standard culture conditions, MG63 cells exhibited the lowest division rate over 72 h (Figure S8H), suggesting the lowest cholesterol requirement, which could partially explain why these cells presented the lowest sensitivity to FR194738. Our findings infer that SQLE inhibition by FR194738 could be benign for cells with low SQLE protein expression or low GRs.

**FIGURE 8:**
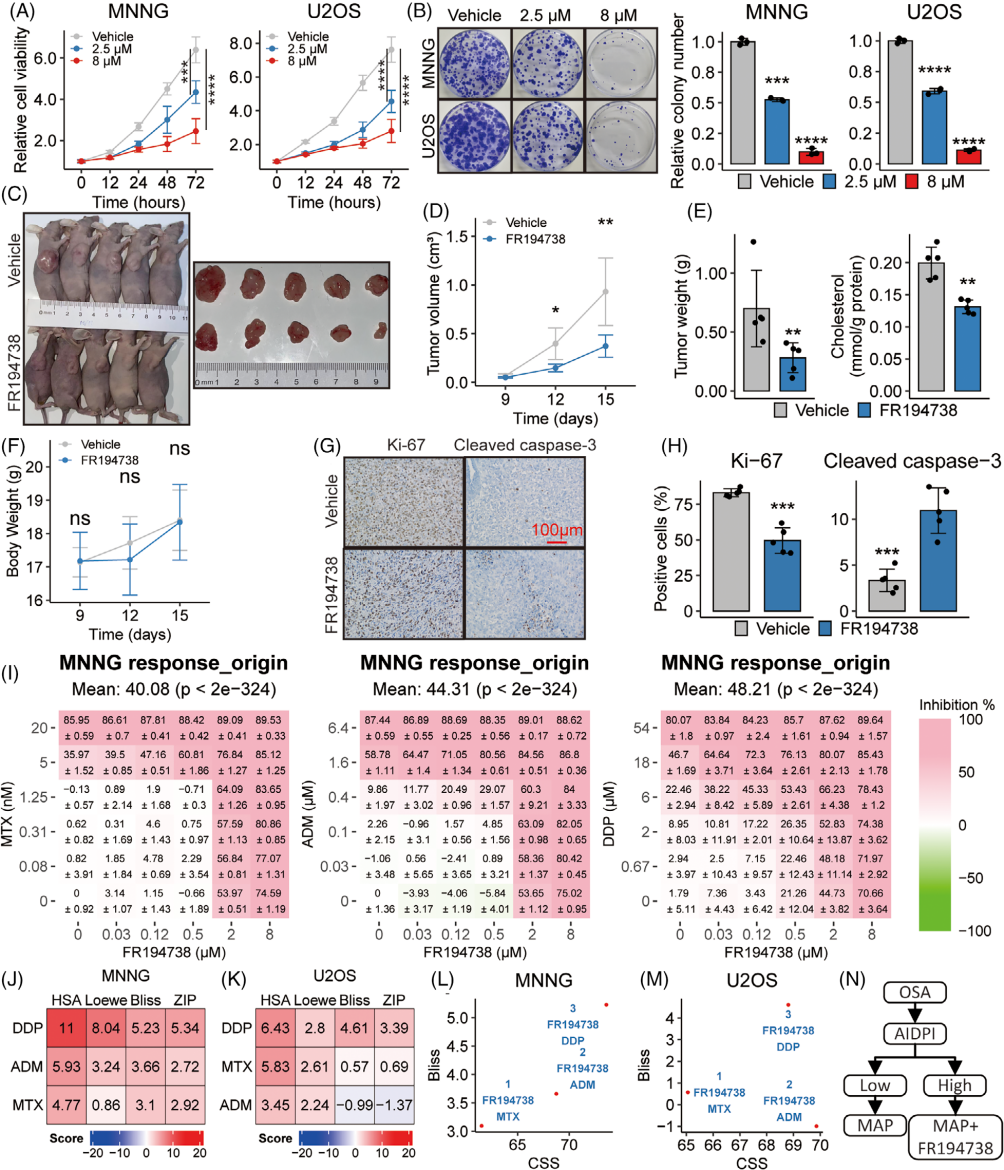
Pharmacologic squalene epoxidase (SQLE) inhibition suppresses osteosarcoma (OSA) progression and enhances chemotherapy efficacy. (A and B) Experiments demonstrate the effects of two concentrations of FR194738 compared to the vehicle control on cell proliferation (A) and colony formation ability (B). (C) Images of isolated tumours from the subcutaneous xenograft models treated with vehicle or FR194738. (D) Tumour growth curves of the indicated groups. (E) Weight and cholesterol levels of tumour masses are presented in bar plots. (F) Body weight curves of the indicated groups. (G) Representative images of immunohistochemistry for Ki‐67 and cleaved caspase‐3 staining in tumour sections. (H) Bar plots contrast the percentage of Ki‐67‐ and cleaved caspase‐3‐positive cells. (I) Heat maps provide dose–response visualizations of MNNG to a spectrum of drug combinations. (J and K) Heat maps present synergy scores when coadministering FR194738 with the indicated agents against the indicated OSA cell lines. (L–M) Scatter plots show both sensitivity metrics and synergistic metrics in the indicated OSA cell lines. (N) Schematic diagram displays the possible way of applying artificial intelligence‐derived prognostic index (AIDPI) in clinical practice. ns: *p* > .05; *p < .05; **p < .01; ***p < .001; ****p < .0001, by Tukey HSD test for all line plots, Student's *t*‐test for all bar plots and adjusted using Holm's method for multiple comparisons.

In subsequent analyses, FR194738 induced dose‐dependent suppression of both proliferation and colony formation in U2OS and MNNG cells (Figure 8A,B). After FR194738 treatment, a congruent decline in intracellular cholesterol was observed (Figure S8I). Efficacy assessment in vivo utilizing MNNG‐bearing xenograft models corroborated our in vitro observations. FR194738 stunted tumour growth and cholesterol content without inducing weight loss in the subjects (Figure 8C–F). Dissected tumour weight comparisons revealed a TGI rate of 59.67% (Figure 8E). Furthermore, IHC analyses for Ki‐67 and cleaved caspase‐3 confirmed reduced cell proliferation and heightened apoptosis in OSA xenografts post‐FR194738 administration (Figure 8G,H), highlighting the therapeutic potential of SQLE pharmacological inhibition for OSA.

Building on the observation that *SQLE* silencing deactivated the FAK/PI3K/Akt/mTOR signalling pathway, which, when inhibited, increases OSA cells’ sensitivity to MTX, ADM and DDP,^45^ we hypothesized that FR194738 synergizes with these first‐line chemotherapy drugs in OSA. We thus assessed cellular responses to various drug combinations using MNNG (Figure 8I) and U2OS (Figure S8J). Most synergy scores suggested potent synergistic effects (Figure 8J,K). Alongside synergy scores, we evaluated the efficacy of drug combinations using the combination sensitivity score (CSS).^62^ Both CSS values and synergy scores were plotted for all examined drug combinations. Remarkably, the combination of FR194738 and DDP stood out as the best combination (Figure 8L,M). These findings suggest that the efficacy of chemotherapy, especially DDP, could be improved in OSA patients through SQLE targeting via FR194738. In line with our findings, we advocate for the clinical potential of the AIDPI in stratifying OSA patients. Although traditional therapies could suffice for low‐AIDPI patients, high‐AIDPI patients might benefit from an integrated approach incorporating traditional therapies and SQLE inhibitors such as FR194738 (Figure 8N).

## 4 DISCUSSION

The 5‐year survival rate for OSA remains below 70%,^63^ highlighting the fact that many OSA patients do not respond to standard therapies. By identifying these high‐risk patients and tailoring supplementary treatments, outcomes may be improved. In pursuit of this objective, we built multiple prognostic models by using 101 machine‐learning algorithm combinations based on the whole‐transcriptome profiles of 254 OSA biopsy samples. Subsequently, the 12‐gene AIDPI, derived from the combination of the CoxBoost and GBM algorithms, emerged as superior.

CoxBoost can be used to fit a Cox proportional hazards model by componentwise likelihood‐based boosting.^64^ This approach is particularly effective for models involving many predictors. In our initial modelling phase, CoxBoost was used primarily for dimensionality reduction and variable screening. However, the model's direct application yielded modest accuracy, achieving an average C‐index of .768. We also used the gbm package, which extends Friedman's GBM,^65^ covering some regression methods, including Cox proportional hazards partial likelihood. A significant challenge with GBM is its lack of inherent dimensionality reduction capabilities, coupled with a heightened risk of overfitting. Consequently, although the GBM‐only model exhibited a high C‐index of .941 in the training set, its performance dropped in the validation and independent testing sets, with C‐indices of .775 and .695, respectively. To address these challenges, we combined the strengths of CoxBoost and GBM. Initially, CoxBoost was employed to select 12 prognosis‐associated genes from the initial set of 18 genes. This step was crucial in reducing the risk of overfitting. Subsequently, the final model was established using GBM, which significantly enhanced accuracy. This combined approach proved to be the most effective, outperforming other models in our study.

In recent years, numerous OSA prognostic signatures have emerged. However, many of these signatures have been derived from limited cohorts or have focused solely on specific gene sets, such as the unfolded protein response gene set,^11^ while ignoring the influence of other biological processes on OSA progression. Our 12 AIDPI genes were selected from the entire transcriptomic profile and encompassed multiple biological processes. *MYC* is a pivotal oncogene whose overexpression is recurrently linked to unfavourable OSA outcomes.^10^ *SQLE* and *MUC1*, associated with cholesterol biosynthesis and mucin production, respectively, are connected to OSA's proliferation and migration.^16^, ^66^ Furthermore, the augmented expression of *CORT* and *MCAM* might also drive OSA progression.^67^, ^68^ *FDPS* promotes prostate cancer progression,^69^ hinting at its potential unfavourable role in OSA. The observed anti‐OSA effects of anti‐TPD52 antiserum in vivo warrant attention.^70^ Conversely, *PMEPA1*, which suppresses the TGF‐beta signalling pathway,^71^ might act as an OSA suppressor, especially given this pathway's oncogenic roles in OSA.^72^ The promoting role of the WNT signalling pathway in OSA^73^ also makes *CTNNBIP1*, a WNT pathway antagonist,^74^ a potential OSA suppressor. Elevated *GLIPR1* expression correlates with macrophage differentiation and displays anti‐OSA effects via miR‐16.^75^ EVI2B protein has been identified in CD8^+^ T cells within OSA tissues.^76^ The FPR1 protein is present in multiple immune cells.^77^ These results indicate that our AIDPI, established by using these 12 genes, reflects the influence of multiple biological processes associated with OSA progression, which could explain its increased predictive precision over clinicopathological markers and existing OSA signatures.

Apart from introducing an AIDPI for stratifying high‐risk OSA patients, we also identified SQLE as a therapeutic target for these patients. SQLE, which oxidizes squalene to (*S*)‐2,3‐epoxy squalene, is a pivotal rate‐limiting enzyme in cholesterol synthesis.^15^ Cholesterol modulates several pathways via cholesterol/sphingolipid‐rich lipid rafts in cell membranes.^57^ Both heightened endogenous cholesterol synthesis and elevated circulating cholesterol promoted cancer's resistance to multiple drugs, spurring interest in repurposing lipid‐modifying drugs for cancer treatment.^78^ Although drugs targeting lipids, such as HMG‐CoA reductase inhibitors (e.g. simvastatin), have shown promise against OSA in preclinical settings,^79^, ^80^ their efficacy in humans remains uncertain.^81^ Previous studies indicated ectopic expression of *SQLE* contributes to enhanced growth and elevated cholesterol levels in the U2OS cell line.^82^ Conversely, the inhibition of SQLE protein has demonstrated an enhanced efficacy of chemotherapy in colorectal cancer,^83^ and silencing *SQLE* decreases cholesterol levels and inhibits the proliferation, colony formation and migration of both U2OS and Saos2 cell lines.^16^, ^82^ However, the mechanisms behind these effects and the in vivo anti‐OSA potential of SQLE inhibitors remain under‐investigated.

In this study, we reported that *SQLE* expression was upregulated in the high‐AIDPI patients, especially in the OSA cells, and high *SQLE* expression was associated with poor prognoses in OSA cohorts. Interestingly, we linked *SQLE* overexpression in OSA to its concurrent amplification with *MYC*, which was previously identified but overlooked.^55^, ^84^ Additionally, we found that *SQLE* knockdown in OSA cell lines led to reduced proliferation and increased apoptosis, attributed to cholesterol reduction and subsequent suppression of the FAK/PI3K/Akt/mTOR pathway, which aligns with prior findings that cholesterol depletion triggers apoptosis through FAK inactivation, internalization of lipid rafts and reduced cell adhesion.^85^ The osteoid‐rich ECM uniquely produced by OSA offers not only a structural scaffold for cells but also functional stimulation.^49^ Focal adhesions, comprising integrins, FAK, and other proteins, are hubs that relay stimulating signals from ECM to the cytoplasm, regulating cell activities such as survival, proliferation and migration.^86^, ^87^ The importance of the FAK/PI3K/Akt/mTOR pathway in OSA is accentuated by a genomic study,^88^ and experimental findings including that CXCL1 promotes OSA lung metastasis via the CXCR2/FAK/PI3K/Akt pathway and that anti‐OSA effects can be achieved by inhibiting the FAK/PI3K/Akt pathway.^89^, ^90^ In this study, the same OSA progression‐associated pathways, including PI3K–Akt signalling, focal adhesion and ECM‐receptor interaction, were simultaneously enriched in KEGG enrichment analyses based on DEGs between low‐ and high‐AIDPI patients and DEGs of U2OS following silencing *SQLE*. Crucially, silencing *SQLE* was proven to deactivate the FAK/PI3K/Akt/mTOR pathway, indicating that targeting SQLE protein has considerable therapeutic potential for high‐risk OSA patients.

Furthermore, we selected the SQLE protein inhibitor, FR194738, for an in‐depth study due to its significant efficacy and specificity. FR194738 showed a promising ability to suppress OSA progression in animal models without significant side effects. Its synergistic potential was evident when combined with agents from the MAP regimen, especially with DDP. This synergistic effect echoes a recent report that attributed DDP resistance to SQLE mRNA upregulation, emphasizing that SQLE inhibition can bolster DDP's therapeutic effects in head‐and‐neck squamous cell carcinoma.^91^

These results suggest that, in clinical practice, we can procure biopsy samples from newly diagnosed OSA patients and conduct RNA‐seq analysis to elucidate their transcriptomic profiles. Subsequently, we can harmonize these profiles with the meta‐OSA cohort and employ our established Shiny app to compute the AIDPI. Based on the AIDPI cutoff value, these OSA patients can be categorized into either low‐ or high‐AIDPI groups. Conventional therapies could be adequate for patients in the low‐AIDPI group, and those patients in the high‐AIDPI group could potentially benefit from integrating traditional treatments with SQLE inhibitors.

Nevertheless, the limitations of our study deserve mention. Our retrospective approach has its drawbacks, emphasizing the need for subsequent prospective trials to validate the predictive capacity of the AIDPI. Beyond cholesterol reduction, it remains to be explored whether SQLE inhibition induces anti‐OSA effects through squalene accumulation, a phenomenon observed in neuroendocrine tumours.^92^ The observed inverse correlation between SQLE mRNA and CD4^+^ T‐cell infiltration, coupled with improved outcomes of SQLE inhibition alongside immune checkpoint blockades in glioblastoma,^93^ suggests a need to scrutinize the effects of SQLE and FR194738 on OSA's immune response. Although our findings, combined with those of prior animal studies,^94^ indicate FR194738's safety and efficacy, rigorous evaluation through additional preclinical models, such as OSA patient‐derived xenografts and transgenic mouse models,^95^ remains necessary.

## 5 CONCLUSIONS

In summary, this research introduced AIDPI as a potential tool for identifying a high‐risk subset of OSA patients and revealed that SQLE protein is a metabolic vulnerability for these patients. Through cholesterol reduction and disruption of the FAK/PI3K/Akt/mTOR signalling pathway, SQLE inhibition using FR194738 offered a promising therapeutic avenue, representing a potential supplementary treatment for high‐risk OSA patients.

## AUTHOR CONTRIBUTIONS

Yongjie Wang and Chunlin Zhang conceptualized and designed the study. Yongjie Wang executed the computational analyses and visualization. In vitro and in vivo validation experiments were carried out by Yongjie Wang, Xiaolong Ma, Enjie Xu and Zhen Huang. Yongjie Wang drafted the manuscript. Chen Yang guided analyses of scRNA‐seq data. Chunlin Zhang, Yang Dong and Kunpeng Zhu conducted overall study supervision.

## CONFLICT OF INTEREST STATEMENT

No potential conflicts of interest were disclosed.

## ETHICS STATEMENT AND CONSENT TO PARTICIPATE

This study was approved by the Ethics Committee of Shanghai Tenth People's Hospital and performed in strict accordance with the Declaration of Helsinki. All participants or their relatives signed informed consent documentation. All animal procedures complied with guidelines established by the Animal Experimental Ethics Committee of Shanghai Tenth People's Hospital (Reference number: SHDSYY‐2020‐3018) and followed the approved protocols.

## Supporting information

Supporting information

## Data Availability

RNA‐seq data generated in this study have been deposited in GEO (GSE245562). All custom codes supporting the findings of this study are available from the corresponding author upon reasonable request.
