# Supplementary material for: Identifying squalene epoxidase as a metabolic vulnerability in high‐risk osteosarcoma using an artificial intelligence‐derived prognostic index
Source: Clin Transl Med. 2024 Feb 19;14(2):e1586. doi: 10.1002/ctm2.1586 (PMC10875711; doi:10.1002/ctm2.1586)
Supplement: Supplementary file 1 — Supporting information [file CTM2-14-e1586-s001.docx]

## Supplementary Data


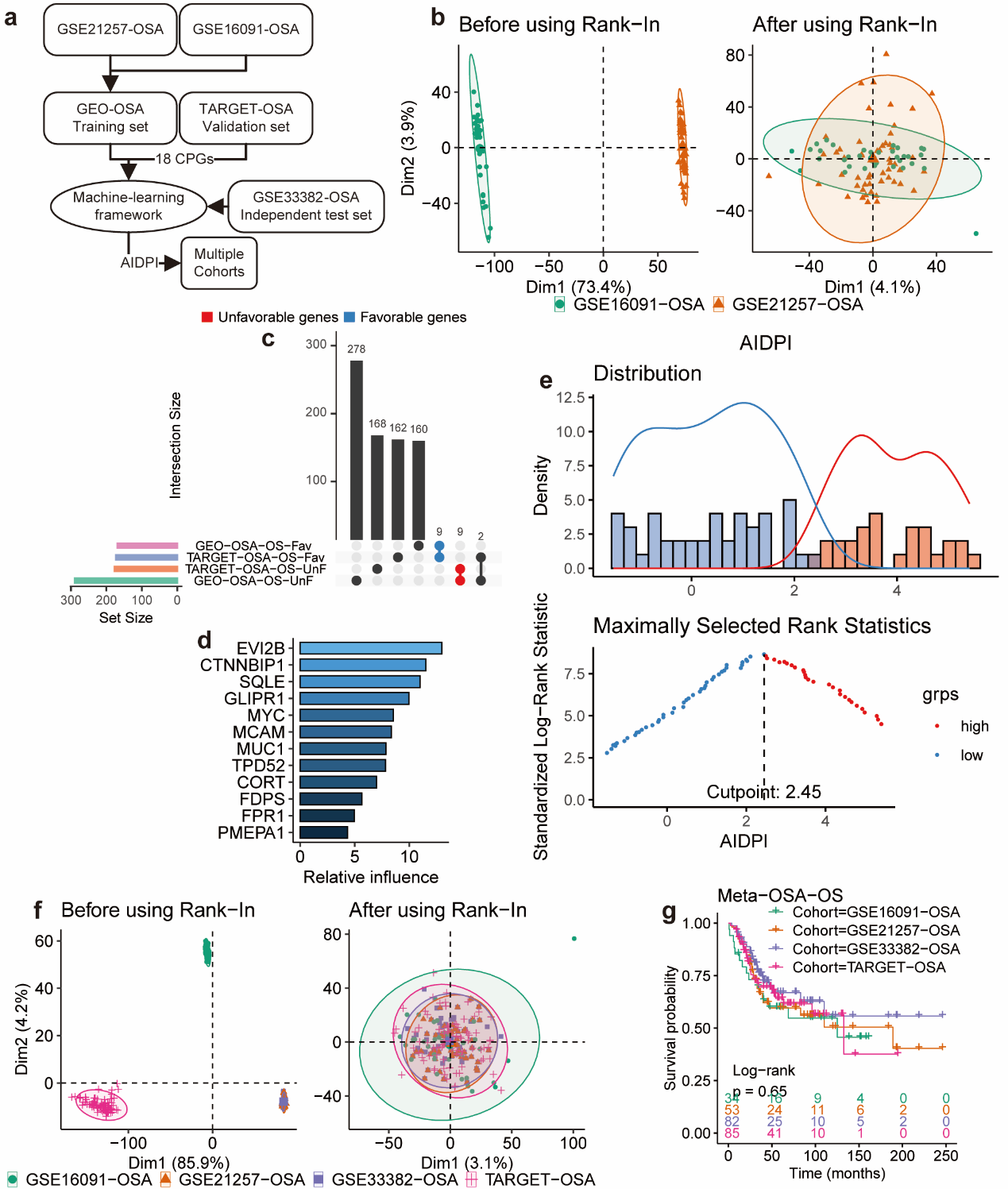


**Figure S1. (a)** Workflow illustrating the development and validation of AIDPI. **(b)** PCA plots of the GEO-OSA cohort highlight samples’ dispersion before and after removing the batch effect. **(c)** An UpSet diagram depicts overlapping prognostic genes identified in both GEO-OSA and TARGET-OSA. **(d)** A bar plot presents chosen genes alongside their corresponding relative influence determined by the GBM algorithm. **(e)** Distribution pattern of AIDPI values with designated optimal threshold for group stratification in GEO-OSA. **(f)** PCA plots of the Meta-OSA cohort show samples’ dispersion before and after removing the batch effect. **(g)** Kaplan-Meier survival analysis emphasizes consistent survival probabilities across the quartet datasets that were combined into the Meta-OSA cohort.


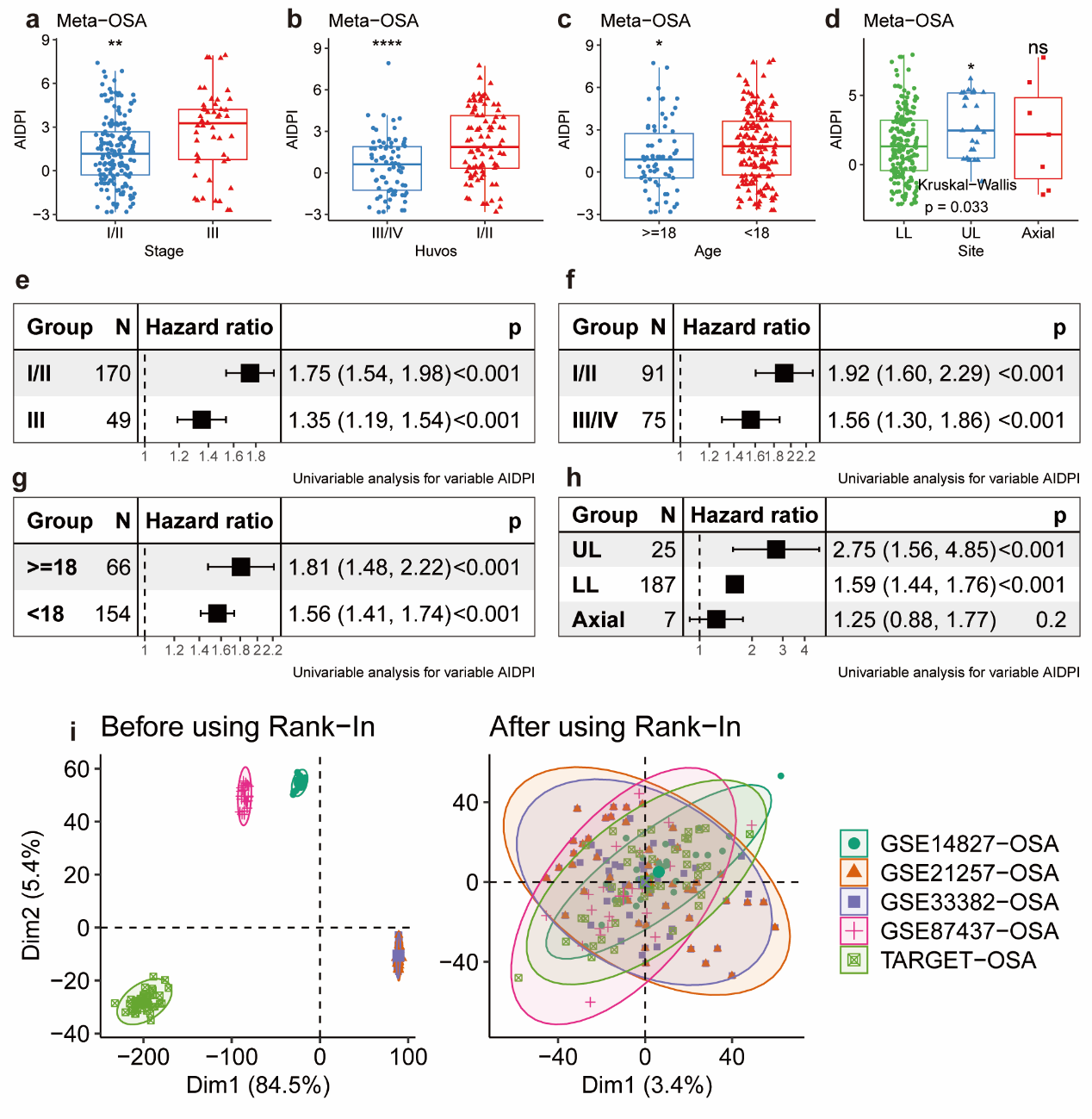


**Figure S2.** **(a-d)** Boxplots contrast AIDPI values across various clinical classifications, including MSTS stages (a), Huvos grades (b), age (c), and primary tumor sites (d). **(e-h)** Forest plots show the results of univariate Cox regression analysis for AIDPI across clinical subgroups defined by MSTS stage (e), Huvos grade (f), age (g), and primary tumor site (h). **(i)** PCA plots of the OSA-Huvos cohort show samples’ dispersion before and after removing the batch effect. ns: p > 0.05; *p < 0.05; **p < 0.01; ****p < 0.0001, by Wilcoxon rank-sum test with adjustment using Holm’s method (a-d).


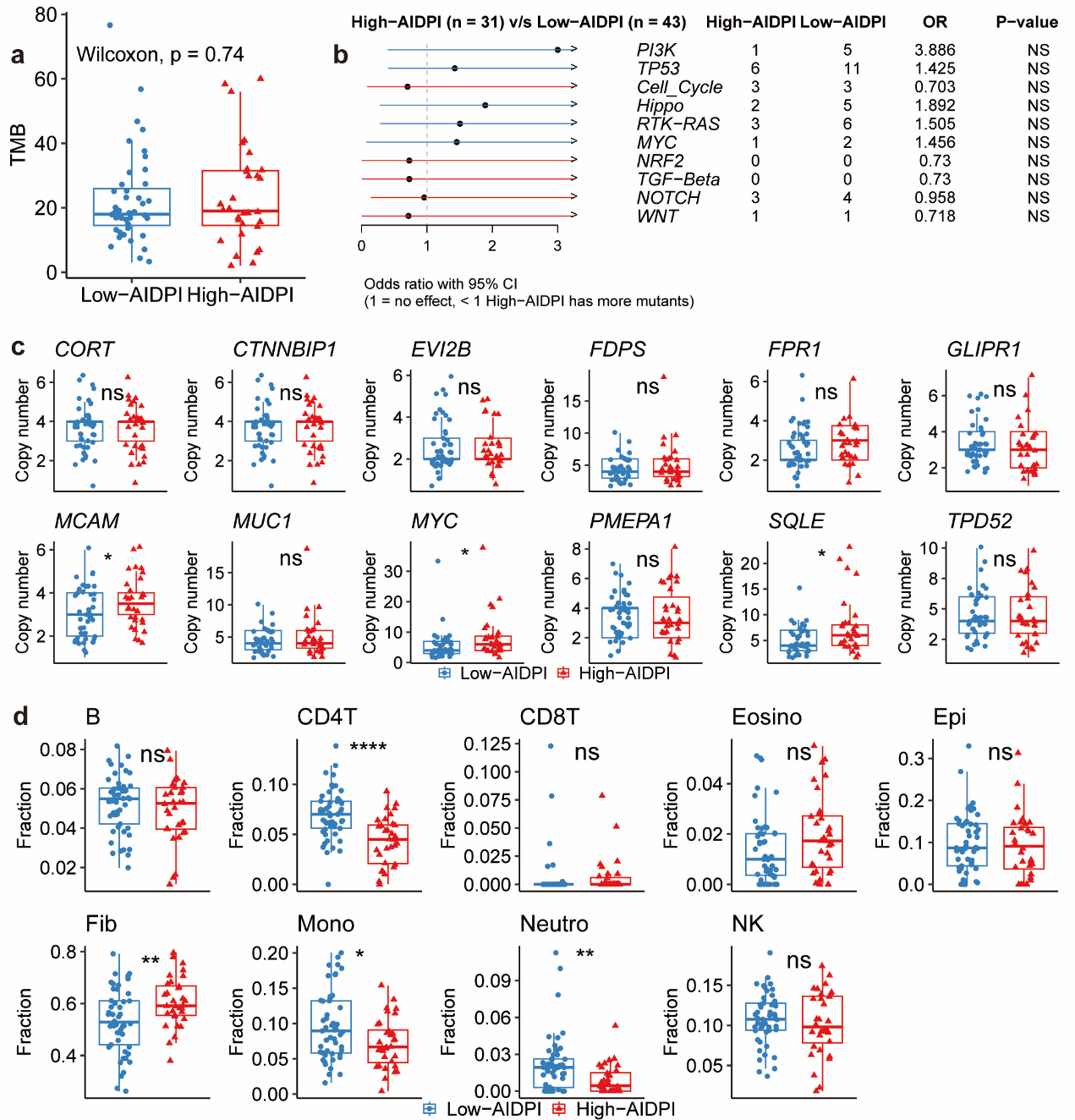


**Figure S3. (a)** A boxplot contrasts TMB between the two AIDPI groups. **(b)** A forest plot contrasts mutation frequencies of genes in specific pathways between the two groups, NS: p > 0.05 by Fisher test. **(c)** Boxplots contrast DNA copy numbers of AIDPI genes between the two groups. **(d)** Boxplots contrast estimated fractions of immune and stromal cells in tumor microenvironments between the two groups. ns: p > 0.05; *p < 0.05; **p < 0.01; ****p < 0.0001, by Wilcoxon rank-sum test.


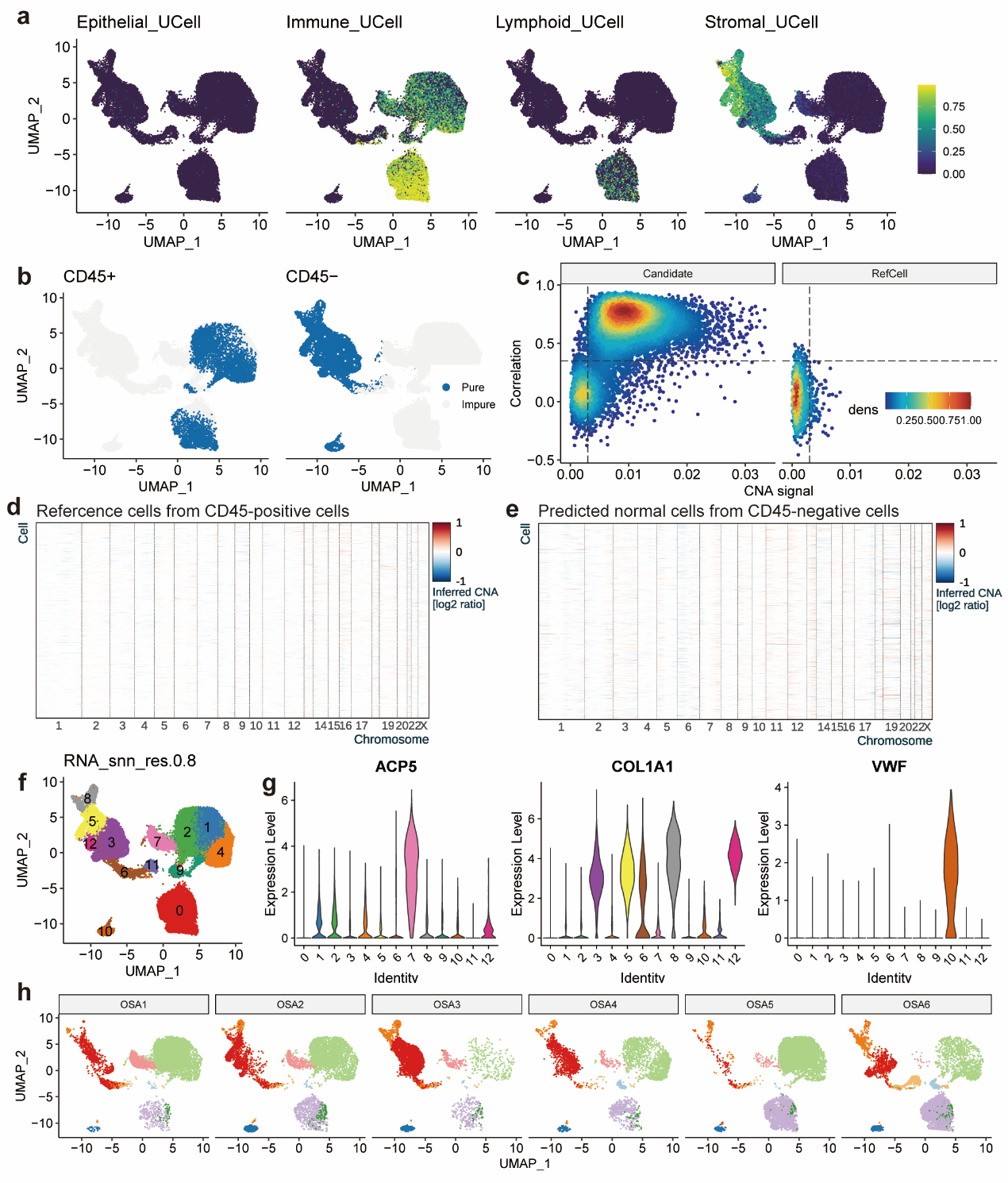


**Figure S4.** **(a)** Uniform Manifold Approximation and Projection (UMAP) visualizations derived from scRNA-seq data illustrate the initial annotations. **(b)** UMAP plots display pure CD45-positive and pure CD45-negative cells. **(c)** Scatter plots are employed to define thresholds that separate tumor cells from candidate cells. **(d-e)** Chromosomal CNAs are inferred based on the mean relative expression spanning windows of 150 genes among reference cells (d) and presumed normal cells (e). **(f)** UMAP illustrates the identified 12 main cell clusters. **(g)** Violin plots depict the normalized expressions of three marker genes: ACP5 (indicative of osteoclasts), VWF (representative of endothelial cells), and COL1A1 (confirming stromal cells). **(h)** UMAP plots for all single cells across all OSA biopsy samples.


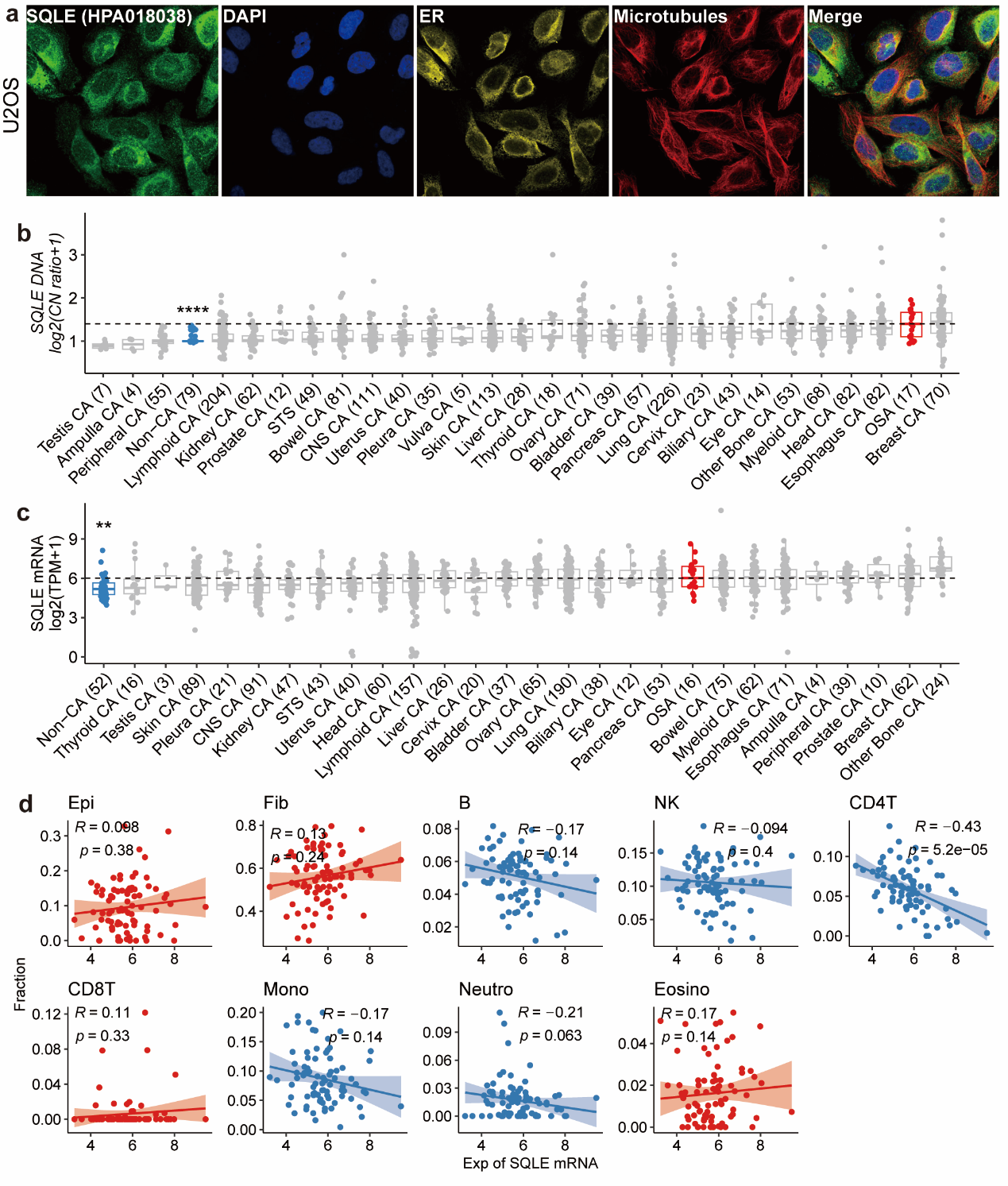


**Figure S5. (a)** Immunofluorescence images depicting subcellular localization of SQLE in U2OS. **(b-c)** Boxplots display SQLE DNA copy number (b) and mRNA expression (c) in cancer cell lines and non-cancer cells (Non-CA). Comparisons were performed between OSA and Non-CA, **p < 0.01; ****p < 0.0001 by Wilcoxon rank-sum test. **(d)** Scatter plots elucidate relationships between SQLE mRNA and deduced fractions of infiltrating immune and stromal cells. CN ratio: gene copy number ratio in a single cell relative to the average across all cells. ER: endoplasmic reticulum.


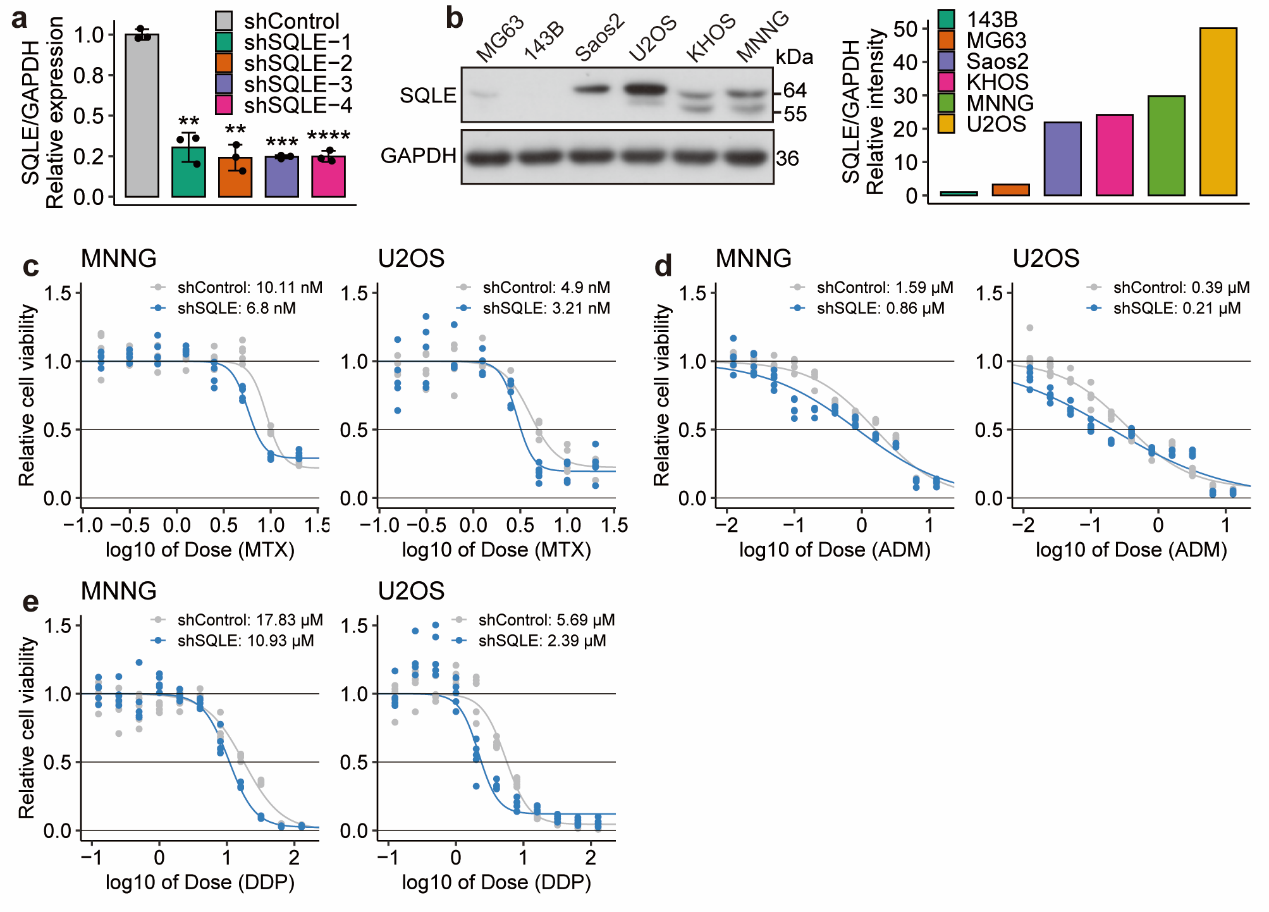


**Figure S6**. **(a)** The qRT-PCR assessed the SQLE mRNA expression levels to validate the knockdown efficacy of the four shRNAs. **(b)** Western blotting analysis was employed to ascertain SQLE protein expression across six OSA cell lines, with histogram plots illustrating the relative intensities normalized to 143B. **(c-e)** The IC_50_ values of the indicated drugs were calculated across the indicated cell groups. **p < 0.01; ***p < 0.001; ****p < 0.0001, by Student’s t-test with adjustment using Holm’s method.


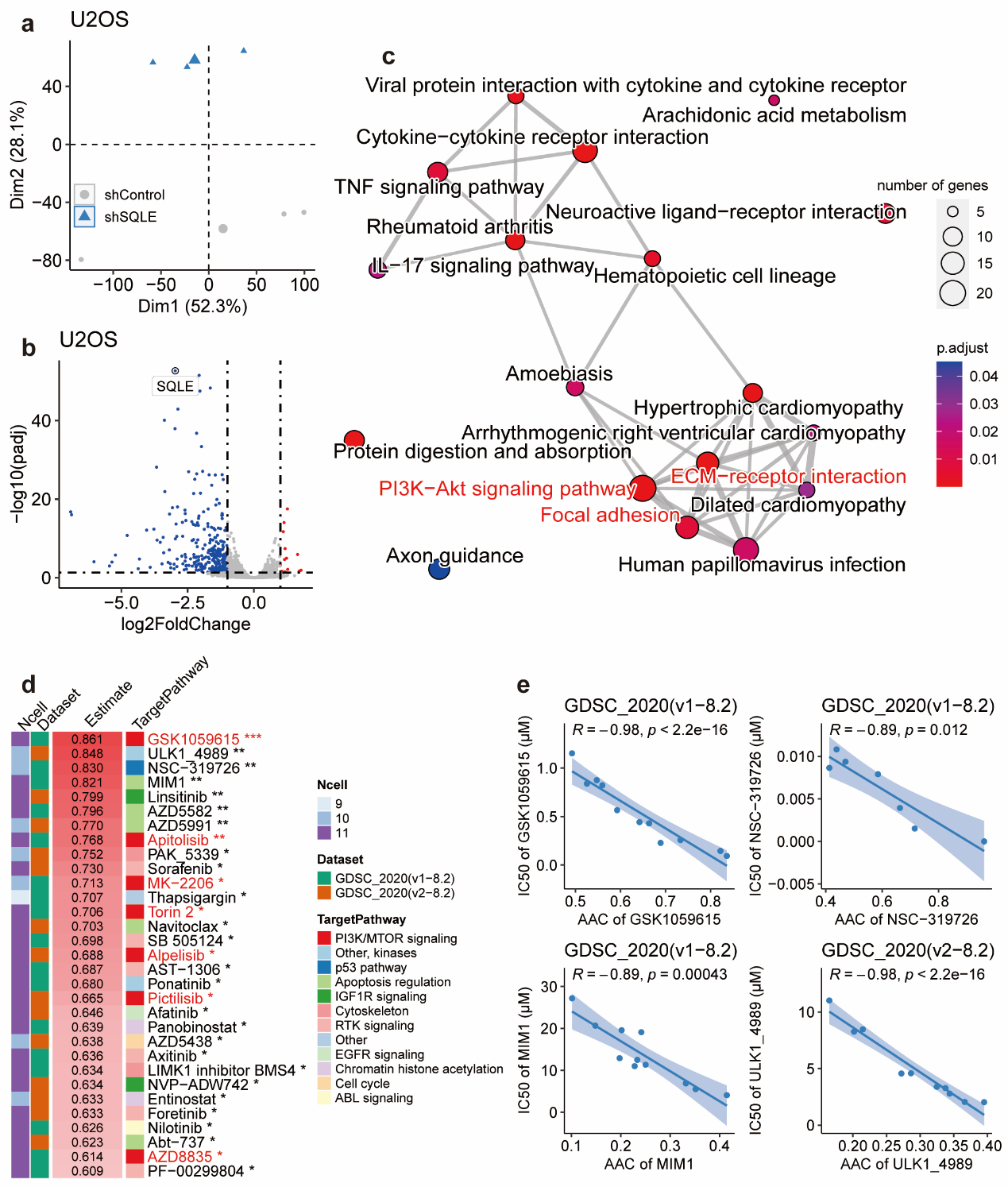


**Figure S7.** **(a)** PCA depicts transcriptomic variations between U2OS-shControl and U2OS-shSQLE cells. **(b)** A volcano plot highlights a significant downregulation of SQLE in the U2OS-shSQLE group. **(c)** An enrichment map arranges enriched terms into a network, connecting the terms that have shared genes. **(d)** A heatmap shows drugs whose sensitivities in OSA cell lines correlate positively with SQLE mRNA expression levels. **(e)** Scatter plots present relationships between IC_50_ values and AACs of four specific drugs in OSA cell lines. Ncell: number of OSA cell lines detected. AAC: area above the dose-response curve. *p < 0.05; **p < 0.01; ***p < 0.001; ****p < 0.0001.


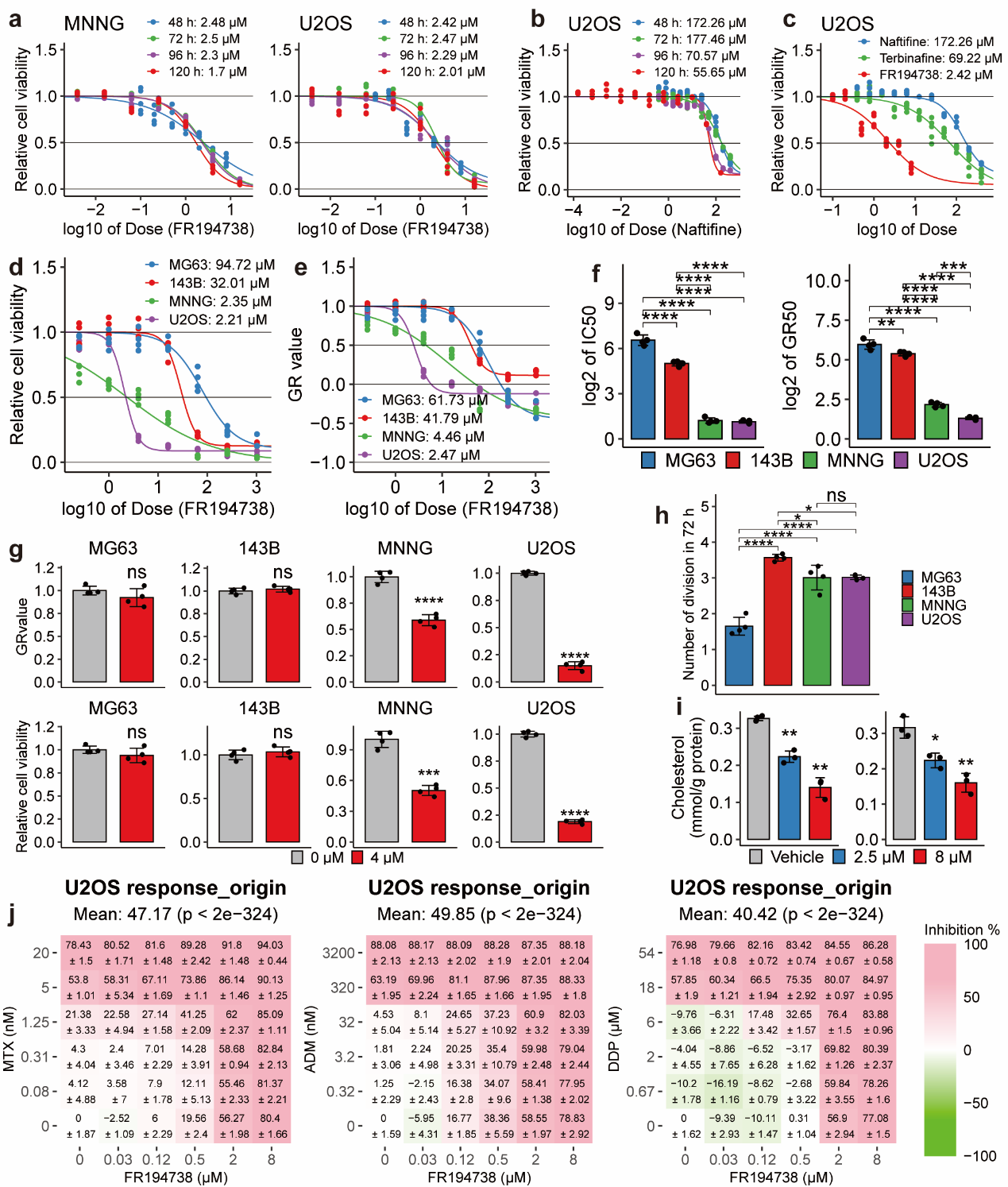


**Figure S8.** **(a-b)** IC_50_ values are determined for two SQLE inhibitors given to specified cells over the indicated duration. **(c)** IC_50_ values of naftifine, terbinafine, and FR194738 are compared when administered to U2OS for 48 hours. **(d-e)** IC_50_ (d) and GR_50_ (e) values of FR194738 are assessed for indicated cells over 72 hours. **(f)** Bar charts represent a comparison of log2-transformed IC_50_ and GR_50_ values of FR194738 across indicated OSA cell lines. **(g)** Bar charts show the growth rate (GR) and cell viability of indicated OSA cell lines post-treatment with varying concentrations of FR194738 for 72 hours. **(h)** Bar charts depict division numbers of specific cells under standard culture conditions over 72 hours. **(i)** Assays illustrate the impact of two dosages of FR194738 versus a vehicle control on intracellular cholesterol levels. **(j)** Heatmaps offer a visual representation of the dose-response relationship in U2OS exposed to an array of drug combinations. ns: p > 0.05; *p < 0.05; **p < 0.01; ***p < 0.001; ****p < 0.0001, by two-sided Student’s t-test adjusted using Holm’s method.

**Table S1.** Information on the signatures mentioned in this research

| **Signature** | **Gene** | **Coefficient** | **Type** | **PMID** |
| --- | --- | --- | --- | --- |
| Chen SJ | ADAR | -0.011308 | mRNA | 35568866 |
|  | CSTF2 | 0.03583 |  |  |
|  | WTAP | -0.01465 |  |  |
| Chen XY | AC009779.2 | -0.08566 | lncRNA | 34425868 |
|  | AC026271.3 | 0.116683 |  |  |
|  | AC135178.5 | 0.134252 |  |  |
|  | AL1333380.1 | 0.120016 |  |  |
|  | AL591895.1 | -0.03249 |  |  |
|  | AP005264.1 | 0.21311 |  |  |
|  | LINC01857 | -0.35896 |  |  |
|  | LINC02454 | 0.065331 |  |  |
|  | LPPAS2 | -0.22239 |  |  |
| Fu YC_1 | DCN | -4.46 | mRNA | 33816483 |
|  | P4HA1 | 4.01 |  |  |
| Fu YC_2 | HSPB1 | 0.44 | mRNA | 35598382 |
|  | IER3 | -0.34 |  |  |
| Goh TS | AP3S2 | -0.427391347 | Mix | 30604867 |
|  | C17orf55 | 0.129505697 |  |  |
|  | C18orf58 | 0.28766188 |  |  |
|  | C1orf168 | -2.401943448 |  |  |
|  | C6orf168 | 0.00310058 |  |  |
|  | C7orf23 | 0.275248885 |  |  |
|  | FAM81A | 0.259209349 |  |  |
|  | HIBCH | 0.330606619 |  |  |
|  | INSM2 | 1.931018781 |  |  |
|  | LOC645201 | -2.381779602 |  |  |
|  | LOC646507 | 1.672096966 |  |  |
|  | LOC647666 | 0.711271707 |  |  |
|  | LOC652635 | -2.311831681 |  |  |
|  | LOC653451 | -0.562000733 |  |  |
|  | MEST | -0.36075991 |  |  |
|  | MPG | 1.220928154 |  |  |
|  | RPUSD1 | 0.233439738 |  |  |
|  | TGFB2 | -0.03496169 |  |  |
|  | TRAP1 | 0.004627901 |  |  |
| Gong HL | CDK3 | 2.0065 | mRNA | 37102261 |
|  | MYC | 0.9253 |  |  |
|  | UHRF2 | 0.6018 |  |  |
|  | STC2 | 0.4926 |  |  |
|  | COL5A2 | 0.418 |  |  |
|  | MMD | -1.0587 |  |  |
|  | EHMT2 | -1.2564 |  |  |
| He Y | ELFN1-AS1 | 0.4704 | lncRNA | 35494024 |
|  | LINC00837 | 0.1634 |  |  |
|  | AL669970.3 | 0.4341 |  |  |
|  | OLMALINC | 0.2696 |  |  |
|  | AC005332.4 | -1.1261 |  |  |
|  | AC023157.3 | -2.223 |  |  |
| Hong JJ | ARHGEF2 | 0.01366902 | mRNA | 35576501 |
|  | ATF4 | 0.3539663 |  |  |
|  | BAK1 | -0.030721347 |  |  |
|  | BCL10 | -0.194735056 |  |  |
|  | BNIP3 | 0.440217961 |  |  |
|  | CRIP1 | 0.019610751 |  |  |
|  | EYA2 | -0.022000565 |  |  |
|  | MAGEA3 | -0.146158789 |  |  |
|  | PDK1 | 0.048849779 |  |  |
|  | PDK2 | -0.248592614 |  |  |
|  | PLEKHF1 | -0.043632439 |  |  |
|  | PML | -0.157323635 |  |  |
|  | PSMD10 | -0.057405651 |  |  |
|  | PTGIS | 0.128940864 |  |  |
|  | PTPN1 | -0.143609316 |  |  |
|  | RNF34 | -0.406556251 |  |  |
|  | RPS3 | 0.062098512 |  |  |
|  | TERT | 0.526581575 |  |  |
|  | TGFBR1 | -0.090918483 |  |  |
|  | TRIM32 | -0.027423747 |  |  |
|  | UNC5B | 0.047796417 |  |  |
| Hu C | FCGR2B | -0.766 | mRNA | 32854645 |
|  | GFAP | 0.702 |  |  |
|  | MPP7 | 0.387 |  |  |
| Hu H | BTBD10 | -1.862613 | mRNA | 37007130 |
|  | DLX1 | 0.2978399 |  |  |
|  | MRTFA | -0.9252084 |  |  |
|  | PLCD3 | 0.1514946 |  |  |
|  | RFX3 | 1.0547832 |  |  |
| Jiang F | EFNA1 | 0.74 | mRNA | 34337075 |
|  | MAFF | 0.62 |  |  |
|  | P4HA1 | 0.63 |  |  |
|  | STC2 | 0.31 |  |  |
| Jiang JX | AC124798.1 | 0.065762645 | lncRNA | 36388161 |
|  | AC006033.2 | -0.109837226 |  |  |
|  | AL450344.2 | -0.115274786 |  |  |
|  | AL512625.2 | -0.1512732 |  |  |
|  | LINC01060 | 0.021443894 |  |  |
|  | LINC008374 | 0.179123438 |  |  |
|  | AC004943.2 | -0.08468658 |  |  |
|  | AC064836.3 | 0.212243158 |  |  |
|  | AC100821.2 | 0.054191142 |  |  |
| Jiang RY | ATG7 | -0.9427 | mRNA | 36254233 |
|  | CBS | 0.4763 |  |  |
|  | MUC1 | 0.2692 |  |  |
|  | PEBP1 | -0.7295 |  |  |
|  | SOCS1 | -0.4567 |  |  |
| Lei T | ALOX15B | 0.902212994 | mRNA | 34506683 |
|  | ATG7 | -0.668153678 |  |  |
|  | CBS | 0.090367621 |  |  |
|  | DPP4 | -0.019240524 |  |  |
|  | EGLN1 | 0.182768936 |  |  |
|  | G6PD | -0.065538143 |  |  |
|  | MUC1 | 0.178257302 |  |  |
|  | MYC | 0.336193028 |  |  |
|  | PEBP1 | -0.687714327 |  |  |
|  | PGD | -0.028691641 |  |  |
|  | SLC39A8 | -0.061396094 |  |  |
|  | SOCS1 | -0.317892139 |  |  |
| Li JY | ATG4A | -1.155 | mRNA | 34604383 |
|  | MYC | 0.478 |  |  |
| Lie F | RP11_326A19.5 | -0.6503 | Pseudogene | 31146489 |
|  | RP4_706A16.3 | 0.636 |  |  |
|  | RPL11-551L14.1 | -0.4327 |  |  |
|  | RPL7AP28 | -1.1344 |  |  |
| Liu BF_1 | GAS5 | 0.351230255 | lncRNA | 36105232 |
|  | LINC00963 | -0.885405813 |  |  |
|  | AL133371.2 | -0.714856133 |  |  |
|  | AC087623.1 | 0.493281992 |  |  |
|  | AC007383.1 | 0.467979376 |  |  |
|  | LBX2AS1 | -0.410460488 |  |  |
|  | VPS9D1-AS1 | 0.380008102 |  |  |
|  | AC027348.1 | -2.348112573 |  |  |
| Liu BF_2 | LINC02315 | -2.66553 | lncRNA | 36313774 |
|  | RUSC1-AS1 | 1.585861 |  |  |
|  | TIPARP-AS1 | 1.343192 |  |  |
|  | UNC5B-AS1 | 1.60588 |  |  |
| Liu H | FTO | -1.066 | mRNA | 33952279 |
|  | IGF2BP1 | 0.271 |  |  |
| Liu WM | PSMC4 | -1.239 | mRNA | 34342651 |
|  | CXCL13 | 0.219 |  |  |
|  | GBP2 | -0.65 |  |  |
|  | CCL2 | 0.507 |  |  |
|  | PPARG | -0.77 |  |  |
|  | CD79A | -0.681 |  |  |
|  | BCL10 | -1.525 |  |  |
|  | FPR1 | -0.483 |  |  |
|  | BMP8B | 0.481 |  |  |
|  | CORT | 0.548 |  |  |
|  | JAG2 | -0.735 |  |  |
|  | STC2 | 0.69 |  |  |
|  | MTNR1B | 0.186 |  |  |
|  | TNFRSF21 | -0.929 |  |  |
| Liu Z | RP2 | -0.8445 | mRNA | 35282067 |
|  | PHB | 0.7205 |  |  |
|  | MYO6 | 0.8332 |  |  |
|  | MLH1 | -0.9115 |  |  |
|  | CSNK2B | -0.6136 |  |  |
|  | RPL37A | 0.6677 |  |  |
|  | CEBPA | -0.5183 |  |  |
| Liu ZY | GALNT14 | 0.5412 | mRNA | 36569869 |
|  | IFI44 | -0.5294 |  |  |
|  | ISLR | -0.2011 |  |  |
|  | ITGB5 | -0.5158 |  |  |
| Long H | MYCT1 | 0.21 | mRNA | 36151259 |
|  | BNIP3L | 0.19 |  |  |
|  | LRP1 | 0.13 |  |  |
|  | OPTN | 0.11 |  |  |
|  | TRIP6 | 0.02 |  |  |
|  | ATF4 | 0.005 |  |  |
|  | TNFRSF1A | -0.07 |  |  |
|  | CLTCL1 | -0.94 |  |  |
| Lv YG | AIM1 | -0.1043 | mRNA | 36275664 |
|  | EVI2B | -0.1913 |  |  |
|  | PRKACB | -0.1502 |  |  |
|  | TCEA3 | 0.2488 |  |  |
| Ma YB | HUWE1 | -0.675 | mRNA | 35874628 |
|  | MYC | 0.585 |  |  |
|  | EIF4G2 | -1.375 |  |  |
|  | USP10 | -0.729 |  |  |
|  | KIF25 | 0.911 |  |  |
|  | TRIM8 | 0.595 |  |  |
|  | CASP1 | -1.218 |  |  |
|  | STUB1 | -0.732 |  |  |
|  | CRYBA1 | 1.391 |  |  |
| Moha. AG | YRDC | 0.8638 | mRNA | 35559393 |
|  | ZC3HAV1 | 0.8321 |  |  |
|  | TERT | 0.8503 |  |  |
|  | RBM34 | 1.0753 |  |  |
|  | TLR8 | -1.4991 |  |  |
|  | IGF2BP2 | 0.3633 |  |  |
|  | NXT2 | -0.2851 |  |  |
| Ni SM | AL033384.2 | 1.743 | lncRNA | 36129020 |
|  | AL031775.1 | -1.495 |  |  |
|  | AC110995.1 | -1.785 |  |  |
|  | LINC00565 | -2.283 |  |  |
| Pan RS | AOC3 | 0.43972 | mRNA | 36189307 |
|  | CDK6 | -0.44907 |  |  |
|  | COL22A1 | 0.307745 |  |  |
|  | RNASE6 | -0.67038 |  |  |
| Qi W | BNIP3 | 0.3316 | mRNA | 34335109 |
|  | ATG4A | -0.1571 |  |  |
|  | BAK1 | -0.0114 |  |  |
|  | CALCOCO2 | -0.192 |  |  |
|  | CCL2 | -0.0457 |  |  |
|  | DAPK1 | -0.0938 |  |  |
|  | EGFR | -0.0738 |  |  |
|  | FAS | -0.0734 |  |  |
|  | GRID2 | -0.1609 |  |  |
|  | ITGA3 | -0.0313 |  |  |
|  | MYC | 0.2591 |  |  |
|  | RAB33B | -0.0593 |  |  |
|  | USP10 | -0.1215 |  |  |
|  | WIPI1 | -0.0467 |  |  |
| Qi ZP | NUBP1 | -0.2216 | mRNA | 33520450 |
|  | ARHGAP25 | -0.0503 |  |  |
|  | APBB1IP | -0.0429 |  |  |
|  | PEF1 | -0.023 |  |  |
|  | USP11 | -0.0123 |  |  |
|  | FUCA1 | -0.0104 |  |  |
|  | S100A13 | 0.0019 |  |  |
|  | DLG4 | 0.0088 |  |  |
|  | RHBDL2 | 0.0144 |  |  |
|  | BNIP3 | 0.017 |  |  |
|  | CORO6 | 0.0425 |  |  |
|  | KIF25 | 0.057 |  |  |
|  | RAD23A | 0.0741 |  |  |
|  | FATE1 | 0.0862 |  |  |
|  | STC2 | 0.0895 |  |  |
|  | COL13A1 | 0.091 |  |  |
|  | SLC38A5 | 0.1003 |  |  |
|  | MYC | 0.1635 |  |  |
|  | CGREF1 | 0.2105 |  |  |
|  | CORT | 0.2504 |  |  |
| Shao HY | TPM1 | -0.3072 | mRNA | 35300639 |
|  | SERPINE2 | 0.2282 |  |  |
|  | TUBA1A | -0.0369 |  |  |
|  | DCN | -0.0618 |  |  |
|  | S100A13 | 0.2319 |  |  |
|  | ST3GAL4 | 0.1904 |  |  |
|  | LOXL1 | -0.113 |  |  |
|  | TUBB | -0.0527 |  |  |
|  | PEF1 | -0.0465 |  |  |
|  | PSMD10 | -0.0549 |  |  |
|  | FAM207A | 0.3118 |  |  |
| Shi DY | MYC | 0.4998 | mRNA | 34894177 |
|  | COL13A1 | 0.2715 |  |  |
|  | UHRF2 | 0.3338 |  |  |
|  | MT1A | 0.2558 |  |  |
|  | ACTB | -0.4997 |  |  |
|  | GBP1 | -0.2012 |  |  |
| Shi Y | MYC | 1.063 | mRNA | 31898371 |
|  | CPE | 0.8383 |  |  |
|  | LY86 | -1.371 |  |  |
| Su ZX | GPI | 0.0026 | mRNA | 36275679 |
|  | MGEA5 | 0.017 |  |  |
|  | OGT | 0.042 |  |  |
|  | PGM3 | 0.057 |  |  |
|  | UAP1 | 0.008 |  |  |
| Tang HJ | EPHX2 | 0.705 | mRNA | 37090691 |
|  | FDPS | 0.478 |  |  |
|  | GBP1 | -0.35 |  |  |
|  | MMD | -0.726 |  |  |
|  | ZYX | -0.815 |  |  |
| Tian KS | RAI14 | -0.13626991 | mRNA | 35435626 |
|  | MAF | -0.13438968 |  |  |
|  | CLEC5A | -0.30713757 |  |  |
|  | TIAL1 | 0.09256004 |  |  |
|  | CENPJ | 0.05059066 |  |  |
| Wan Y | CGREF1 | 0.235 | mRNA | 34488541 |
|  | DNAI1 | 0.457 |  |  |
|  | ZDHHC23 | 0.652 |  |  |
| Wang QH | STC2 | 0.141522237 | mRNA | 34747719 |
|  | TNFRSF11B | 0.098754475 |  |  |
|  | CORT | 0.086711381 |  |  |
|  | IL22 | 0.084649801 |  |  |
|  | GAL | 0.069818606 |  |  |
|  | PSMC4 | -0.013177704 |  |  |
|  | CD79A | -0.014491145 |  |  |
|  | SEMA3E | -0.017349815 |  |  |
|  | IGLV1-51 | -0.021744664 |  |  |
|  | CMTM1 | -0.0494132 |  |  |
|  | SSTR1 | -0.054281399 |  |  |
|  | TNFRSF21 | -0.057043444 |  |  |
|  | TMPRSS6 | -0.065932207 |  |  |
|  | FGFRL1 | -0.074868654 |  |  |
|  | IGKV1D-33 | -0.075750338 |  |  |
|  | IL7 | -0.078764237 |  |  |
|  | TRAV9-2 | -0.110319916 |  |  |
|  | SDC3 | -0.118469971 |  |  |
|  | IL13RA2 | -0.125900341 |  |  |
|  | GCG | -0.183788709 |  |  |
|  | PPARG | -0.205915021 |  |  |
| Wang XX | ATF4 | 0.705 | mRNA | 36439512 |
|  | ATM | 0.503 |  |  |
|  | HILPDA | 0.616 |  |  |
|  | MUC1 | 0.323 |  |  |
|  | CBS | 0.417 |  |  |
|  | MT1G | 0.238 |  |  |
|  | ARNTL | -0.969 |  |  |
|  | PML | -0.553 |  |  |
| Wen CK | COCH | 0.3 | mRNA | 33281116 |
|  | MYOM2 | 0.41 |  |  |
|  | PDE1B | -2.1 |  |  |
| Wu B | UGT3A2 | 0.7661 | mRNA | 33832059 |
|  | SAXO2 | -1.0487 |  |  |
|  | PLEKHG1 | -0.884 |  |  |
|  | DSCR8 | 0.6846 |  |  |
|  | MYL1 | 0.6977 |  |  |
|  | TMEM125 | -0.8993 |  |  |
|  | GAGE1 | 0.3215 |  |  |
|  | SCN1A | -1.1019 |  |  |
| Wu CW | MYC | 0.8115 | mRNA | 37055822 |
|  | P4HA1 | 0.5565 |  |  |
|  | RAMP1 | 0.4634 |  |  |
|  | TAC4 | 0.3422 |  |  |
| Wu GZ | KCNJ15 | 0.0501 | mRNA | 32448271 |
|  | SLC24A4 | -0.392 |  |  |
|  | ASPA | 0.0661 |  |  |
|  | REM1 | -0.0633 |  |  |
|  | SCARA5 | -0.024 |  |  |
|  | LANCL3 | 0.143 |  |  |
|  | CPA6 | 0.0522 |  |  |
|  | TRH | 0.0592 |  |  |
| Wu ZY | LINC01517 | 0.673727216 | lncRNA | 36066162 |
|  | GAS5 | 0.552563452 |  |  |
| Xie L | DLX2 | 0.4837165 | mRNA | 36814224 |
|  | TERT | 0.832482 |  |  |
|  | EVX1 | 0.8523485 |  |  |
| Xu AK | CKLF | -0.537343156 | mRNA | 34828292 |
|  | DKK1 | 0.312512732 |  |  |
|  | MYC | 0.320496576 |  |  |
| Xu FX | RPS28 | 0.513 | mRNA | 36618348 |
|  | MCAM | 0.701 |  |  |
|  | EN1 | -0.718 |  |  |
|  | TRAM2 | 0.575 |  |  |
|  | VEGFA | 0.467 |  |  |
| Yang F | TERT | 0.1984387 | mRNA | 35504036 |
|  | TRAP1 | 0.61052614 |  |  |
|  | DNM1L | 0.09921504 |  |  |
|  | BAG5 | 0.19947814 |  |  |
|  | PLEKHF1 | -0.30350396 |  |  |
|  | PPP3CB | -0.33883222 |  |  |
| Yang JP | C3orf14 | -0.106872216 | mRNA | 35075228 |
|  | UHRF2 | 0.321564173 |  |  |
|  | DDX26B | 0.334217953 |  |  |
|  | ZFP90 | -0.505473926 |  |  |
|  | FBXL5 | -0.24934174 |  |  |
|  | UBE2L3 | -0.308369838 |  |  |
|  | MYC | 0.250664103 |  |  |
|  | CLTC | -0.356348745 |  |  |
|  | ARX | 0.444234486 |  |  |
|  | CTNNBIP1 | -0.601488248 |  |  |
|  | CORT | 0.220902785 |  |  |
|  | SELPLG | -0.070201073 |  |  |
|  | WDR53 | -0.059234551 |  |  |
|  | SLC16A3 | 0.002731127 |  |  |
|  | MKL2 | -0.028375916 |  |  |
|  | SLC45A4 | -0.156290246 |  |  |
|  | PLD3 | -0.128837662 |  |  |
| Yang K | SNHG12 | 0.115 | lncRNA | 35422168 |
|  | SNHG7 | 0.062 |  |  |
| Yang LY | MUC1 | 0.11735 | mRNA | 36899330 |
|  | MAP3K5 | -0.23479 |  |  |
|  | LURAP1L | -0.19464 |  |  |
|  | HMOX1 | -0.07795 |  |  |
|  | BNI P3 | 0.20553 |  |  |
| Yang MK | P4HA1 | 0.413 | mRNA | 33952718 |
|  | STC2 | 0.435 |  |  |
|  | ABCB6 | 0.946 |  |  |
| Ying Tang | RP1-261G23.7 | 0.01 | lncRNA | 31850493 |
|  | RP11-69E11.4 | 0.01 |  |  |
|  | SATB2-AS1 | 0.02 |  |  |
| Yu SY | RP11-128N14.5 | 0.247734 | lncRNA | 33964903 |
|  | RP11-231\|13.2 | 0.287661 |  |  |
|  | RP5-894D12.4 | 0.294636 |  |  |
|  | LAMA5-AS1 | 0.310018 |  |  |
|  | RP11-346L1.2 | 0.232996 |  |  |
| Yu YY | CXCR3 | -0.2888 | mRNA | 32820615 |
|  | SAA1 | 0.1504 |  |  |
|  | CCL4 | 0.2079 |  |  |
|  | PYY | 0.2266 |  |  |
|  | CXCL9 | 0.4665 |  |  |
|  | CXCL11 | -0.4468 |  |  |
|  | S1PR4 | -0.3696 |  |  |
| Zhang J | AMBRA1 | -3.4704 | mRNA | 34513835 |
|  | MYC | 3.823 |  |  |
|  | VEGFA | 1.4389 |  |  |
| Zhang WB | CBS | 0.4 | mRNA | 35503637 |
|  | ACSL5 | -0.475 |  |  |
|  | DDAH2 | -0.43 |  |  |
|  | PDE4C | 0.654 |  |  |
|  | PNPO | -0.878 |  |  |
| Zhang WS | BNIP3 | 0.6371 | mRNA | 36578780 |
|  | SLC38A5 | 0.3938 |  |  |
|  | SLC5A3 | -0.563 |  |  |
|  | CKMT2 | 0.3709 |  |  |
|  | S100A3 | -0.6061 |  |  |
|  | CXCL11 | -0.4593 |  |  |
|  | PGM1 | -0.7056 |  |  |
| Zhang YQ | LAMA3 | -0.00575 | mRNA | 33308057 |
|  | LGALS1 | -0.00014 |  |  |
|  | SGCG | 0.0565 |  |  |
|  | VEGFA | 0.00151 |  |  |
|  | WNT5A | -0.00199 |  |  |
|  | MATN3 | -0.00069 |  |  |
|  | ANPEP | -0.00117 |  |  |
|  | FUCA1) | -0.03087 |  |  |
|  | FLNA | -0.00017 |  |  |
| Zhang YX | DDN-AS1 | 1.501 | lncRNA | 35844445 |
|  | AC022915.1 | -0.665 |  |  |
|  | AC090559.1 | -0.566 |  |  |
|  | AL512330.1 | 0.435 |  |  |
|  | SENCR | 0.383 |  |  |
|  | AC073073.2 | -1.886 |  |  |
|  | AC004812.2 | -1.03 |  |  |
| Zhang Z_1 | MTDH | 0.731 | mRNA | 36630752 |
|  | ATF4 | 1.275 |  |  |
|  | MAPK1 | 0.9832 |  |  |
|  | ANXA5 | -1.161 |  |  |
|  | PPARG | -0.97 |  |  |
| Zhang Z_2 | ATP6V0D1 | -1.523 | mRNA | 35754801 |
|  | PREB | 0.903 |  |  |
|  | STC2 | 0.586 |  |  |
|  | TSPYL2 | -0.76 |  |  |
| Zhang ZD | CCL5 | -0.579 | mRNA | 33181717 |
|  | CCL8 | -0.156 |  |  |
|  | CCR4 | -0.714 |  |  |
|  | CCR5 | -0.203 |  |  |
| Zheng D_1 | ABCA3 | 0.566 | mRNA | 34368151 |
|  | CTGF | -0.057 |  |  |
|  | AMIGO2 | -5.564 |  |  |
|  | PREB | 0.175 |  |  |
|  | FHIT | 6.429 |  |  |
|  | EXOSC5 | -0.409 |  |  |
| Zheng D_2 | AP003119.2 | 0.6709 | lncRNA | 35186011 |
|  | LINC01816 | 0.7443 |  |  |
|  | AL139289.1 | 1.1434 |  |  |
|  | AC004812.2 | -4.5629 |  |  |
|  | AC005785.1 | 2.9688 |  |  |
|  | AL353804.1 | 0.6036 |  |  |
| Zheng DZ | FPR1 | -0.006181735 | mRNA | 34630511 |
|  | GBP1 | -0.004282512 |  |  |
|  | FUCA1 | -0.007687824 |  |  |
|  | PDK1 | 0.007877602 |  |  |
|  | BNIP3 | 0.003118838 |  |  |
|  | EVI2B | -0.001487156 |  |  |
|  | APBB1IP | -0.025874691 |  |  |
|  | FOLR2 | -0.000566207 |  |  |
|  | COCH | 0.007318869 |  |  |
| Zhong YJ | CBS | 2.442496728 | mRNA | 36313783 |
|  | GLB1 | -2.316478283 |  |  |
|  | HACD | -1.543819577 |  |  |
| Zhou Y | RPS9 | 4.023231 | mRNA | 35836470 |
|  | RPS23 | -2.22729 |  |  |
|  | EIF4A1 | 1.276326 |  |  |
|  | RPL12 | -1.6577 |  |  |
|  | RPL36 | 1.628909 |  |  |
|  | RPL37A | 2.963511 |  |  |
|  | RPL34 | -1.15058 |  |  |
|  | EEF1B2 | -1.61765 |  |  |
|  | RPS8 | 1.872409 |  |  |
|  | RPS28 | -0.96574 |  |  |
|  | RPL10 | 1.556604 |  |  |
|  | RPS24 | 1.05129 |  |  |
|  | RPL35A | 1.484525 |  |  |
|  | RPL11 | -2.61106 |  |  |
|  | RPL21 | 1.896643 |  |  |
|  | RPS27A | 1.278613 |  |  |
|  | RPS12 | -0.85901 |  |  |
|  | RPL13A | -3.5775 |  |  |

**Table S2.** The basic information on the primary public datasets used in this research

| **Dataset** | **Data type** | **Platform** | **Annotation package** |
| --- | --- | --- | --- |
| TARGET | Multi-omics | / | / |
| GSE33382 | Microarray | GPL10295 | illuminaHumanv2.db |
| GSE42352 | Microarray | GPL10295 | illuminaHumanv2.db |
| GSE21257 | Microarray | GPL10295 | illuminaHumanv2.db |
| GSE16091 | Microarray | GPL96 | hgu133a.db |
| GSE14827 | Microarray | GPL570 | hgu133plus2.db |
| GSE87437 | Microarray | GPL570 | hgu133plus2.db |
| GSE99671 | RNA-seq | GPL20148 | / |
| PRJNA698672 | RNA-seq | / | / |
| GSE238110 | RNA-seq | GPL25760 | / |
| PRJNA681896 | scRNA-seq | 10X Genomics | / |
| CCLE | Multi-omics | / | / |

**Table S3.** shRNA targeting sequence

| **Target** | **shRNA number** | **Sequence** |
| --- | --- | --- |
| SQLE | shRNA-1 | ATGAAGTATATGGTTCATTAA |
|  | shRNA-2 | ATGCACCACAGTTTAAAGCAA |
|  | shRNA-3 | TGGGAGTTCAGTACAAGGATA |
|  | shRNA-4 | AAATCATGCTGAACTTATTT |

**Table S4.** Sequences of primer sets used in quantitative RT-PCR

| **Gene** | **Sequence (5’ to 3’)** |
| --- | --- |
| SQLE | TGGCTTCTTCTGGGCCAAAT |
|  | TTCCTTTTCTGCGCCTCCTG |
| GAPDH | GGAGCGAGATCCCTCCAAAAT |
|  | GGCTGTTGTCATACTTCTCATGG |

**Table S5.** The information on the antibodies used in this research

| **Name** | **Source** | **Identifier** | **RRID** |
| --- | --- | --- | --- |
| SQLE antibody | Proteintech | 12544-1-AP | AB_2195888 |
| Squalene epoxidase (H-6) | SCBT | sc-271651 | AB_10708249 |
| Phospho-FAK (Tyr397) Antibody | CST | 3283 | AB_2173659 |
| Anti-FAK Antibody [SR46-04] | HUABIO | ET1602-25 | AB_3069638 |
| Anti-PI 3 Kinase p85 alpha (phospho Y607) antibody | Abcam | ab182651 | AB_2756407 |
| Recombinant Anti-PI 3 Kinase p85 alpha antibody | Abcam | ab191606 | AB_2891324 |
| pan-AKT (phospho T308) antibody | Abcam | ab38449 | AB_722678 |
| pan-AKT antibody | Abcam | ab8805 | AB_306791 |
| Anti-Phospho-mTOR (S2448) Antibody [A5D5] | HUABIO | HA600094 | AB_3071710 |
| Anti-mTOR Antibody | HUABIO | HA500126 | AB_3071224 |
| GAPDH (D16H11) XP Rabbit mAb | CST | 5174 (also 5174P, 5174S, 5174T) | AB_10622025 |
| Anti-rabbit IgG, HRP-linked Antibody | CST | 7074 (also 7074S, 7074V, 7074P2) | AB_2099233 |
| Anti-mouse IgG, HRP-linked Antibody | CST | 7076 (also 7076S, 7076V, 7076P2) | AB_330924 |
| Ki67 antibody-Proliferation Marker | Abcam | ab15580 | AB_443209 |
| Cleaved Caspase-3 (Asp175) Antibody | CST | 9661 (also NYUIHC-314, 9661S, 9661L) | AB_2341188 |

**Table S6.** Demographical characteristics and clinical data of the patients from TMA

|  | **High-SQLE (n = 51)** | **Low-SQLE (n = 26)** | **P-value** |
| --- | --- | --- | --- |
| **Gender** |  |  |  |
| Female | 16 (31.4%) | 11 (42.3%) | 0.485 |
| Male | 35 (68.6%) | 15 (57.7%) |  |
| **Age (years)** |  |  |  |
| Mean (SD) | 26.0 (16.4) | 26.3 (17.3) | 0.934 |
| Median [Min, Max] | 19.3 [8.58, 80.4] | 18.4 [8.16, 76.4] |  |
| **MSTS stage** |  |  |  |
| I/II | 42 (82.4%) | 25 (96.2%) | 0.179 |
| III | 9 (17.6%) | 1 (3.8%) |  |
| **Primary tumor site** |  |  |  |
| Axial | 2 (3.9%) | 0 (0%) | 0.588 |
| LL | 42 (82.4%) | 22 (84.6%) |  |
| UL | 7 (13.7%) | 4 (15.4%) |  |
| **Death** |  |  |  |
| No | 40 (78.4%) | 24 (92.3%) | 0.224 |
| Yes | 11 (21.6%) | 2 (7.7%) |  |
| **Survival time (months)** |  |  |  |
| Mean (SD) | 41.9 (15.5) | 55.7 (14.1) | < 0.001 |
| Median [Min, Max] | 41.5 [0.533, 71.0] | 61.8 [12.5, 69.9] |  |

**Table S7.** The main R packages and web tools used in this study

| **R package/web tools** | **Version/Link** |
| --- | --- |
| TCGAbiolinks | 2.26.0 |
| GEOquery | 2.66.0 |
| oligo | 1.60.0 |
| beadarray | 2.48.0 |
| hgu133a.db | 3.13.0 |
| hgu133plus2.db | 3.13.0 |
| illuminaHumanv2.db | 1.26.0 |
| Rank-In | http://www.badd-cao.net/rank-in/index.html |
| FactoMineR | 2.8 |
| factoextra | 1.0.7 |
| PharmacoGx | 3.2.0 |
| Seurat | 4.4.0 |
| DoubletFinder | 2.0.3 |
| harmony | 1.0.3 |
| scGate | 1.4.1 |
| infercna | https://github.com/jlaffy/infercna |
| survival | 3.5-7 |
| glmnet | 4.1-8 |
| gbm | 2.1.8.1 |
| randomForestSRC | 3.2.2 |
| plsRcox | 1.7.7 |
| stats | 4.2.3 |
| superpc | 1.12 |
| survivalsvm | 0.0.5 |
| CoxBoost | 1.5 |
| timeROC | 0.4 |
| survminer | 0.4.9 |
| compareC | 1.3.2 |
| ezcox | 1.0.4 |
| forestmodel | 0.6.3 |
| regplot | 1.1 |
| rms | 6.7-1 |
| ggDCA | 1.2 |
| pROC | 1.18.4 |
| DESeq2 | 1.38.3 |
| clusterProfiler | 4.7.1.001 |
| msigdbr | 7.5.1 |
| ComplexHeatmap | 2.14.0 |
| maftools | 2.14.0 |
| ChAMP | 2.28.0 |
| IOBR | 0.99.9 |
| EpiDISH | 2.14.1 |
| GRmetrics | 1.24.0 |
| synergyfinder | 3.8.2 |
| flowCore | 2.12.2 |
| flowGate | 1.0.0 |
| ggcyto | 1.28.1 |
| rstatix | 0.7.2 |
| tidyverse | 2.0.0 |
| ggpubr | 0.6.0 |
| patchwork | 1.1.2 |
| RColorBrewer | 1.1-3 |
| Vennerable | 3.1.0.9000 |
| table1 | 1.4.3 |
| Shiny | 1.7.5.1 |
| DynNom | 5.0.2 |
